# Circular RNA circPOLR2A promotes clear cell renal cell carcinoma progression by facilitating the UBE3C-induced ubiquitination of PEBP1 and, thereby, activating the ERK signaling pathway

**DOI:** 10.1186/s12943-022-01607-8

**Published:** 2022-07-15

**Authors:** Zhipeng Xu, Shuqiu Chen, Ruiji Liu, Hui Chen, Bin Xu, Weizhang Xu, Ming Chen

**Affiliations:** 1 Department of Urology, Affiliated Zhongda Hospital of Southeast University, No.87 Dingjiaqiao Road, Nanjing, 210009 People’s Republic of China; 2 Urology Research Center, Southeast University Medical School, No.87 Dingjiaqiao Road, Nanjing, 210009 People’s Republic of China; 3 Department of Radiation Oncology, The First Affiliated Hospital of Nanjing Medical University, No.300 Guangzhou Road, Nanjing, 210029 People’s Republic of China; 4 Department of Urology, Jiangsu Institute of Cancer Research & Jiangsu Cancer Hospital, No.42 Baiziting Road, Nanjing, 210000 People’s Republic of China; 5 Department of Urology, Nanjing Lishui District People’s Hospital, No.86 Chongwen Road, Nanjing, 211200 People’s Republic of China

**Keywords:** CircPOLR2A, PEBP1, UBE3C, Ubiquitination, N6-methyladenosine, YTHDF2, Clear cell renal cell carcinoma

## Abstract

**Background:**

Increasing evidence has demonstrated that circular RNAs (circRNAs) are implicated in cancer progression. However, the aberrant expression and biological functions of circRNAs in clear cell renal cell carcinoma (cRCC) remain largely elusive.

**Method:**

Differentially expressed circRNAs in cRCC were filtered via bioinformatics analysis. Aberrant circPOLR2A expression was validated in cRCC tissues and cell lines via qRT-PCR. Sanger sequencing was used to identify the backsplicing site of circPOLR2A. In vitro and in vivo functional experiments were performed to evaluate the role of circPOLR2A in cRCC malignancy. RNA pull-down, mass spectrometry, RIP, FISH and immunofluorescence assays were used to identify and validate the circPOLR2A-interacting proteins. Ubiquitination modification and interaction between proteins were detected via Co-IP and western blotting. The m6A modification in circPOLR2A was validated by the meRIP assay.

**Results:**

Bioinformatics analysis revealed that circPOLR2A was highly expressed in cRCC tissues and metastatic cRCC tissues. CircPOLR2A expression was associated with tumor size and TNM stage in cRCC patients. In vitro and in vivo functional assays revealed that circPOLR2A accelerated cRCC cell proliferation, migration, invasion and angiogenesis, while inhibiting apoptosis. Further mechanistic research suggested that circPOLR2A could interact with UBE3C and PEBP1 proteins, and that UBE3C could act as a specific ubiquitin E3 ligase for the PEBP1 protein. The UBE3C/circPOLR2A/PEBP1 protein-RNA ternary complex enhanced the UBE3C-mediated ubiquitination and degradation of the PEBP1 protein which could inactivate the ERK signaling pathway. Rescue experiments revealed that the PEBP1 protein was the functional downstream target of circPOLR2A. Furthermore, m6A modification in circPOLR2A was confirmed, and the m6A reader YTHDF2 could regulate circPOLR2A expression.

**Conclusion:**

Our study demonstrated that circPOLR2A modulated the UBE3C-mediated ubiquitination and degradation of the PEBP1 protein, and further activated the ERK pathway during cRCC progression and metastasis. The m6A reader, YTHDF2, regulated circPOLR2A expression in cRCC. Hence, circPOLR2A could be a potential target for the diagnosis and treatment of cRCC.

**Supplementary Information:**

The online version contains supplementary material available at 10.1186/s12943-022-01607-8.

## Introduction

Renal cell carcinoma (RCC), arising from genetic changes, accounts for approximately 2% of all adult malignancies [1]. Among diverse RCC subtypes, clear cell renal cell carcinoma (cRCC) is the most common type [2]. When technically feasible, surgical excision remains the only curative treatment for RCC [3]. However, it is worth noting that 20–30% of patients are diagnosed with metastases due to the difficulty in early detection [4, 5]. Systemic therapy for metastatic renal cell carcinoma (mRCC), including targeted therapy and immunotherapy, has continuously developed over the last two decades, which has improved the prognosis of mRCC [6]. However, the 5-year survival rate for mRCC is still nearly 10% [6–8]. Hence, it is important to identify potential biomarkers and therapeutic targets for the clinical diagnosis and treatment of cRCC.

Circular RNAs (circRNAs) are covalently closed RNA molecules originating from precursor mRNA via noncanonical backsplicing in eukaryotes [9, 10]. Therefore, there is no 5′ cap or 3′ polyadenylation tail (poly-(A) tail) in circRNAs, which endows circRNAs with resistance to exonucleases and higher stability than their linear RNA counterparts [11]. With the development of bioinformatics technology, accumulating studies have shown that dysregulation of circRNAs is involved in the pathogenesis of cancers, including cRCC [12–14]. For example, circCHST15, circESRP1 and circEXOC7 were reported to be novel biomarkers that regulate the progression of cRCC [15–17]. The reported functions of circRNAs have mainly focused on acting as miRNA sponges [18, 19], acting through proteins [20, 21], and translatable circRNAs [22]. Aberrant expression of circRNAs in cancers eventually regulates multiple signaling pathways. For instance, circAXIN1 could activate the Wnt/β-catenin signaling pathway by encoding a novel protein in gastric cancer [23]. Chen et al. reviewed the circRNAs associated with the PI3K/AKT signaling pathway in cancer progression [24]. These findings suggest that circRNAs play an important role in tumorigenesis and progression. Further study on the roles of circRNAs may contribute to understanding the underlying mechanism involved in cRCC progression.

Phosphatidylethanolamine Binding Protein 1 (PEBP1) is a member of the phosphatidylethanolamine-binding protein family [25]. Initially, Yeung et al. reported that PEBP1 could competitively bind to Raf1, which resulted in dissociation of the Raf1-MEK complex and acted as an inhibitor of the Raf1/MEK/ERK pathway [26, 27]. Subsequent studies verified that PEBP1 was downregulated in diverse cancers, which contributed to the malignant biological behavior of cancer cells [28–30]. Some researchers have validated that PEBP1 could act as a metastasis suppressor gene [29, 30]. In addition, PEBP1 is also involved in regulating cell proliferation, mitosis and drug-induced apoptosis [31–33]. Although early work has reported the potential correlation between PEBP1 and the clinicopathological characteristics of cRCC patients [34–36], the function and metabolism of PEBP1 protein remain unknown during cRCC progression.

N6-methyladenosine (m6A), an epigenetic modification of RNA, is involved in RNA metabolism, including transcription splicing, translation and degradation [37]. It was reported that DRACH (D: G/A/U; R: G/A; H: U/A/C) was the consensus motif of m6A [38]. Recent studies have also concentrated on the potential role of m6A modification on circRNAs. Zhou et al. identified thousands of circRNAs related to m6A in different cell types [39]. Translation initiation based on circRNAs could be driven by m6A modification, which required the m6A reader YTH N6-methyladenosine RNA binding protein 3 (YTHDF3) [40]. Specifically, Chen et al. revealed that the m6A reader YTH domain containing 1 (YTHDC1) modulated the cytoplasmic export of circNSUN2 [21]. A recent study confirmed that the expression of circCPSF6 could be inhibited by YTH N6-methyladenosine RNA binding protein 2 (YTHDF2) in a m6A modification-dependent manner [41]. However, the contributions of m6A-modified circRNAs in cRCC remain to be further elucidated.

In the present study, we screened out a circRNA originating from POLR2A, hsa_circRNA_092437 (circBase ID: hsa_circ_0000741), named circPOLR2A, by analyzing the expression profiles of circRNAs in cRCC. Higher expression of circPOLR2A was verified in cRCC compared to matched adjacent normal tissues. We demonstrated that circPOLR2A promoted cRCC progression by mediating the ubiquitination of PEBP1 and activating the Raf1/MEK/ERK pathway. In addition, we further revealed that low expression of YTHDF2 contributed to upregulation of circPOLR2A in cRCC, which indicated that the expression of circPOLR2A was associated with m6A methylation modification. Our work suggested that circPOLR2A could act as an oncogene in cRCC progression and a potential target in the treatment of cRCC.

## Methods

All methods and materials are described in the Supplemental Methods and Materials.

## Results

### Aberrant expression of circPOLR2A in cRCC

**Fig. 1:**
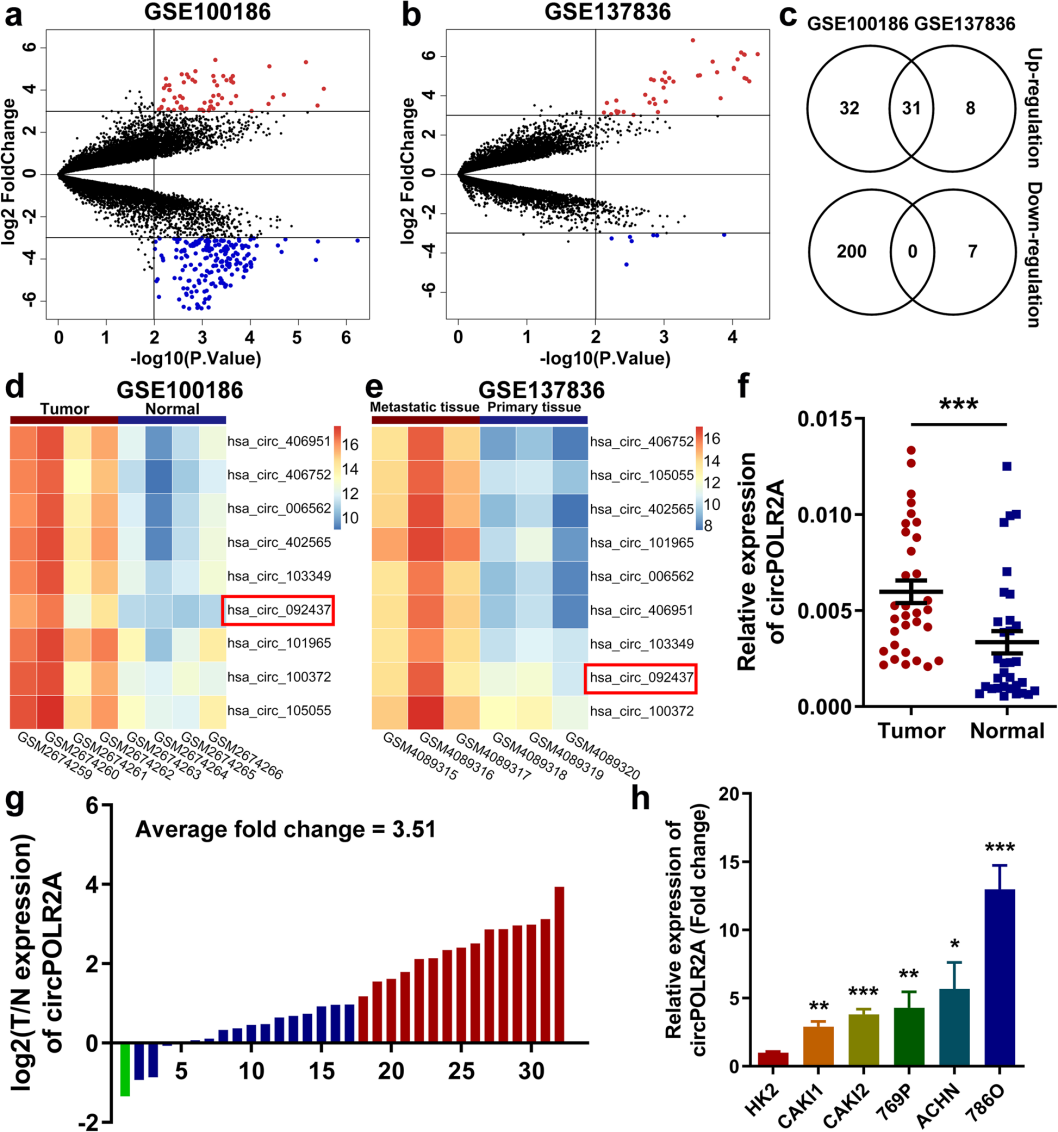
Profiling of dysregulated circRNAs and validation of aberrant expression of circPOLR2A in cRCC. **a**, **b** Volcano plots of GSE100186 (**a**) and GSE137836 (**b**). DE circRNAs were identified with *p* value < 0.01 and log_2_(Fold Change) > 3. The significantly upregulated circRNAs were expressed as red dots, while blue dots represented downregulated circRNAs. **c** Venn diagram of DE circRNAs in two datasets. **d**, **e** Heatmaps of 9 DE circRNAs with high expression in two datasets. **f** The expression of circPOLR2A in 32 paired tissues of cRCC. *P* values were evaluated by paired two-sided t test. **g** Expression ratio of circPOLR2A in 32 paired tissues of cRCC. Red, Log2 (T/N expression) value > 1; blue, 1 ≥ Log2 (T/N expression) value ≥ − 1; Green, Log2 (T/N expression) value < − 1. **h** CircPOLR2A expression was examined in HK2 and cRCC cells via qRT-PCR

To explore the potential role of circRNAs in cRCC, we performed bioinformatics analysis on microarray datasets in the Gene Expression Omnibus (GEO) repository: GSE100186, determining the expression profiles of circRNAs in 4 pairs of cRCC and nontumorous tissues [42]; GSE137836, examining circRNA expression profiles in primary tumor tissues and metastatic tumor tissues [43] (Supplemental Table 1). As shown in volcano plots, we identified differentially expressed (DE) circRNAs with *p* value < 0.01 and log_2_(fold change) > 3 (Fig. 1a and b). Subsequently, Venn diagram suggested that 31 circRNAs were consistently upregulated, while none were synchronously downregulated in the two GEO datasets (Fig. 1c). Considering the feasibility of further experimental research, we analyzed the average expression of each circRNA in two GEO datasets, and selected the candidate circRNAs from highly expressed circRNAs whose expression ranked within the top 10 among DE circRNAs screened out from any GEO dataset (Supplemental Table 2). Finally, 9 circRNAs were selected from the 31 upregulated circRNAs. Heatmap showed the microarray expression of 9 circRNAs in two GEO datasets (Fig. 1d and e).

Next, we determined the 9 circRNAs in 32 pairs of cRCC samples and adjacent normal samples via quantitative reverse transcription PCR (qRT-PCR) with divergent primers. As shown in Fig. 1f, g and Supplemental Fig. 1, circPOLR2A was the most significantly upregulated circRNA among the 9 candidate circRNAs in cRCC samples. In addition, we investigated whether circPOLR2A expression was correlated with clinicopathologic features in the 32 cRCC patients. The results indicated that upregulation of circPOLR2A was significantly associated with tumor size and TNM stage in cRCC patients (Table 1). We further examined the expression of circPOLR2A in the normal human proximal tubular cell line HK2 and diverse human cRCC cell lines. As shown in Fig. 1h, the expression level of circPOLR2A was significantly higher in cRCC cells than in HK2 cells. Among the cRCC cells, 786O and Caki1 cells presented the highest and lowest expression levels of circPOLR2A, respectively. Hence, we chose the two cell lines for further experiments. Taken together, our findings suggested that circPOLR2A was upregulated in cRCC clinical tissues and cell lines, and that its expression was positively correlated with cRCC malignant features.

**Table 1:** The correlation between circPOLR2A expression and clinicopathological characteristics in 32 cRCC patients was evaluated by Fisher's precision probability test via SPSS software

Characteristics	Total (*n*=32)	circPOLR2A expression		*P*-value
		High group	Low group	
**Age(years)**				
≥60	17	9	8	1.000
<60	15	7	8	
**Gender**				
Male	17	8	9	1.000
Female	15	8	7	
**Size(cm)**				
<4.5	14	3	11	0.011*
≥4.5	18	13	5	
**Fuhrman grade**				
1+2	24	10	14	0.220
3+4	8	6	2	
**TNM Stage**				
I	25	9	16	0.007*
II/III	7	7	0	

*p<0.05 Statistically signifcant difference

### Characterization of circPOLR2A in cRCC cells

**Fig. 2:**
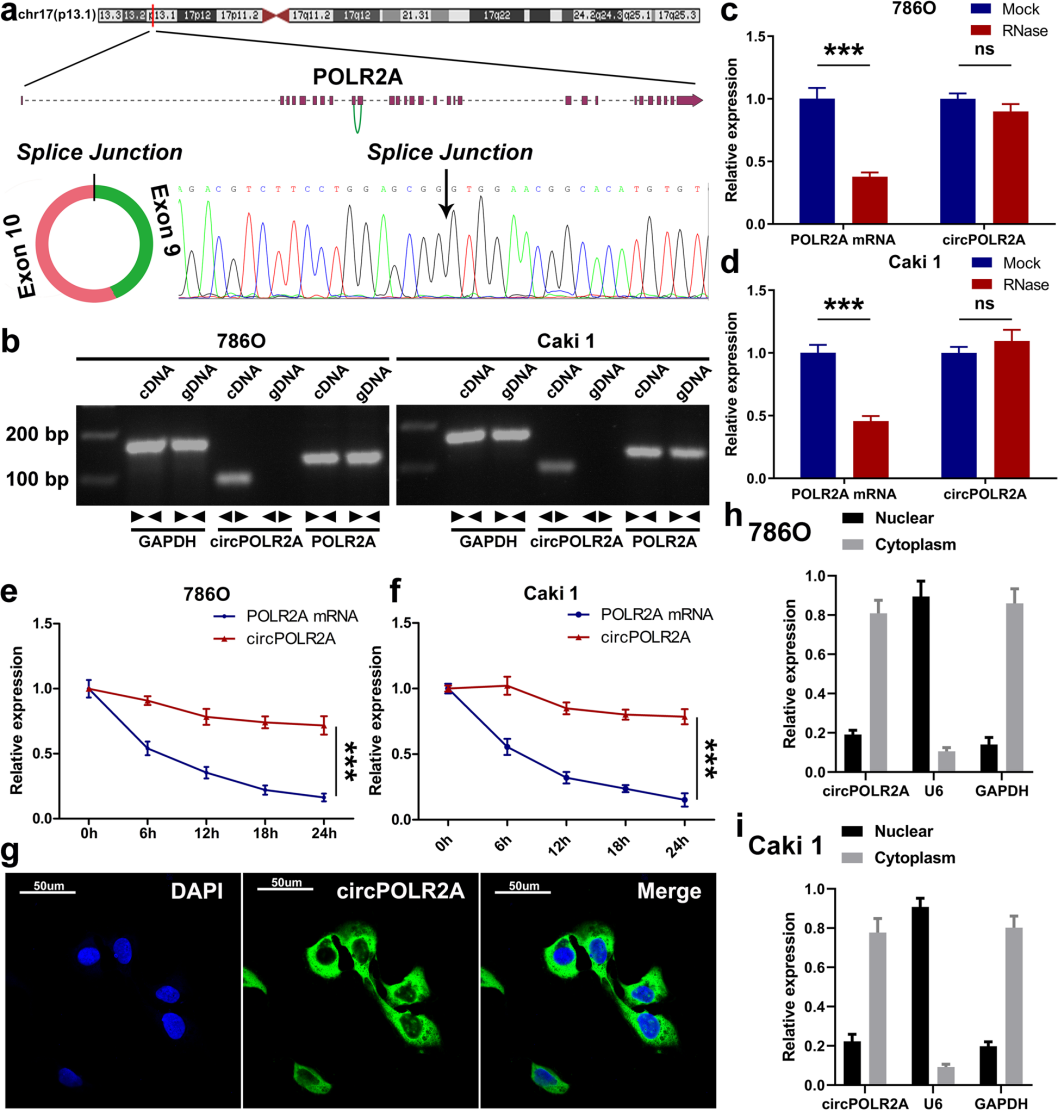
Characterization of circPOLR2A. **a** Schematic illustration of circPOLR2A originating from the POLR2A gene on chromosome 17 (top). The backsplice junction site of circPOLR2A was identified via Sanger sequencing (bottom). **b** The existence of circPOLR2A and POLR2A mRNA in cDNA and gDNA was determined by PCR and electrophoresis analysis. **c**, **d** The abundance of circPOLR2A and POLR2A mRNA after RNase R treatment was detected via qRT-PCR. **e**, **f** The RNA abundance of circPOLR2A and POLR2A mRNA in cRCC cells treated with actinomycin D was determined at different time points. **g** Representative FISH images of circPOLR2A in 786O cells. **h**, **i** Nuclear and cytoplasmic fractionation revealed the level of circPOLR2A in the nucleus and cytoplasm. U6 and GAPDH were used as positive controls in the nucleus and cytoplasm, respectively

circPOLR2A is generated from exons 9–10 of the POLR2A gene on chromosome 17p13.1 (Fig. 2a, top). The mature sequence of circPOLR2A is 336 bp in length. After amplification with divergent primers, the backsplice junction site of circPOLR2A was confirmed via Sanger sequencing (Fig. 2a, bottom). To rule out the possibility of trans-splicing or genomic rearrangements, further experiments were conducted. The convergent primers were designed to amplify POLR2A mRNA, and complementary DNA (cDNA) and genomic DNA (gDNA) from 786O and Caki1 cell lines were used as templates. As shown in Fig. 2b, circPOLR2A was amplified by divergent primers in cDNA, while no amplification product was observed in gDNA. Next, the resistance to digestion by RNase R exonuclease suggested that circPOLR2A harbored a loop structure (Fig. 2c and d). Treatment with actinomycin D (the suppressor of transcription) suggested that circPOLR2A was more stable than POLR2A mRNA (Fig. 2e and f). Furthermore, fluorescence in situ hybridization (FISH) and nuclear and cytoplasmic fractionation verified the predominant location of circPOLR2A in the cytoplasm (Fig. 2g, h and i). These results suggested that circPOLR2A was a stable circRNA expressed in cRCC.

### circPOLR2A exerted effects on cRCC cell proliferation, migration, invasion, and apoptosis in vitro

**Fig. 3:**
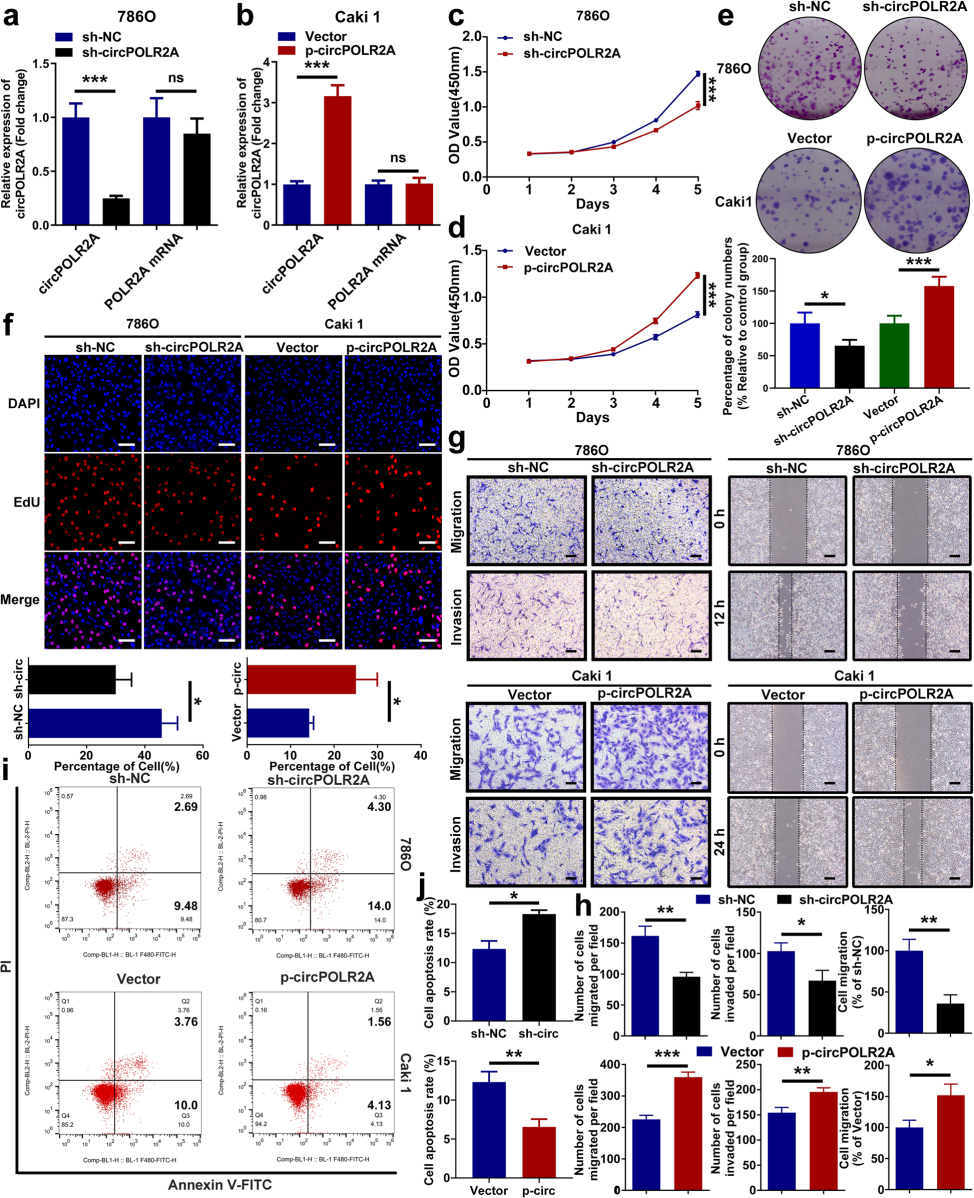
CircPOLR2A promoted malignant biological properties in cRCC cells. **a**, **b** The expression of circPOLR2A and POLR2A mRNA was detected after circPOLR2A knockdown (**a**) or overexpression (**b**) in cRCC cells. **c**, **d** CCK-8 assay indicated the growth rate of cRCC cells transfected with lentivirus for circPOLR2A knockdown (**c**) or overexpression (**d**). Two-way ANOVA was used to evaluate significant differences. **e** Colony formation assay for cRCC cells. **f** EdU incorporation assay was performed to assess DNA synthesis in cRCC cells. Red fluorescence represented EdU-positive cells, while blue fluorescence represented total cells. Scale bar, 100 μm. **g** Cell migration and invasion assays of cRCC cells treated with circPOLR2A knockdown or overexpression. Left, transwell assay, scale bar, 40 μm; right, wound healing assay, scale bar, 100 μm. **h** Statistical analysis of the cell migration and invasion assays. **i**, **j** The effect of circPOLR2A knockdown or overexpression on cell apoptosis via flow cytometry assay

For further research, the knockdown lentivirus and overexpression lentivirus were transfected into 786O cells and Caki1 cells, respectively. As shown in Fig. 3a and b, the expression of circPOLR2A was efficiently regulated by the lentivirus, while the expression of POLR2A mRNA was not targeted. The CCK-8 assay indicated that circPOLR2A overexpression significantly enhanced the cell growth rate in Caki1 cells, while inhibition of circPOLR2A suppressed the cell growth rate in 786O cells (Fig. 3c and d). Next, colony formation assay further confirmed the role of circPOLR2A in cell proliferation (Fig. 3e). In addition, the results of the EdU incorporation assay indicated that 786O cells with circPOLR2A inhibition presented a lower proportion of EdU-positive cells than the control, whereas Caki1 cells with circPOLR2A overexpression showed the opposite effect (Fig. 3f). To investigate the effect of circPOLR2A on cell migration and invasion, transwell assay was performed. As shown in Fig. 3g, left and 3 h, circPOLR2A knockdown weakened migratory and invasive abilities, while ectopic expression of circPOLR2A dramatically accelerated migration and invasion. Moreover, wound healing assay further verified the effect of circPOLR2A on cell migration (Fig. 3g, right and 3 h). Flow cytometry was applied to determine the role of circPOLR2A in cell apoptosis. The 786O cells with circPOLR2A knockdown exhibited a significantly higher rate of apoptosis than the control, while the Caki1 cells with circPOLR2A overexpression showed a lower apoptotic rate than the control (Fig. 3i and j). These data suggested that circPOLR2A was able to enhance cell proliferation, migration and invasion, and suppress apoptosis in cRCC cells.

### circPOLR2A interacted with PEBP1 in cRCC cells

**Fig. 4:**
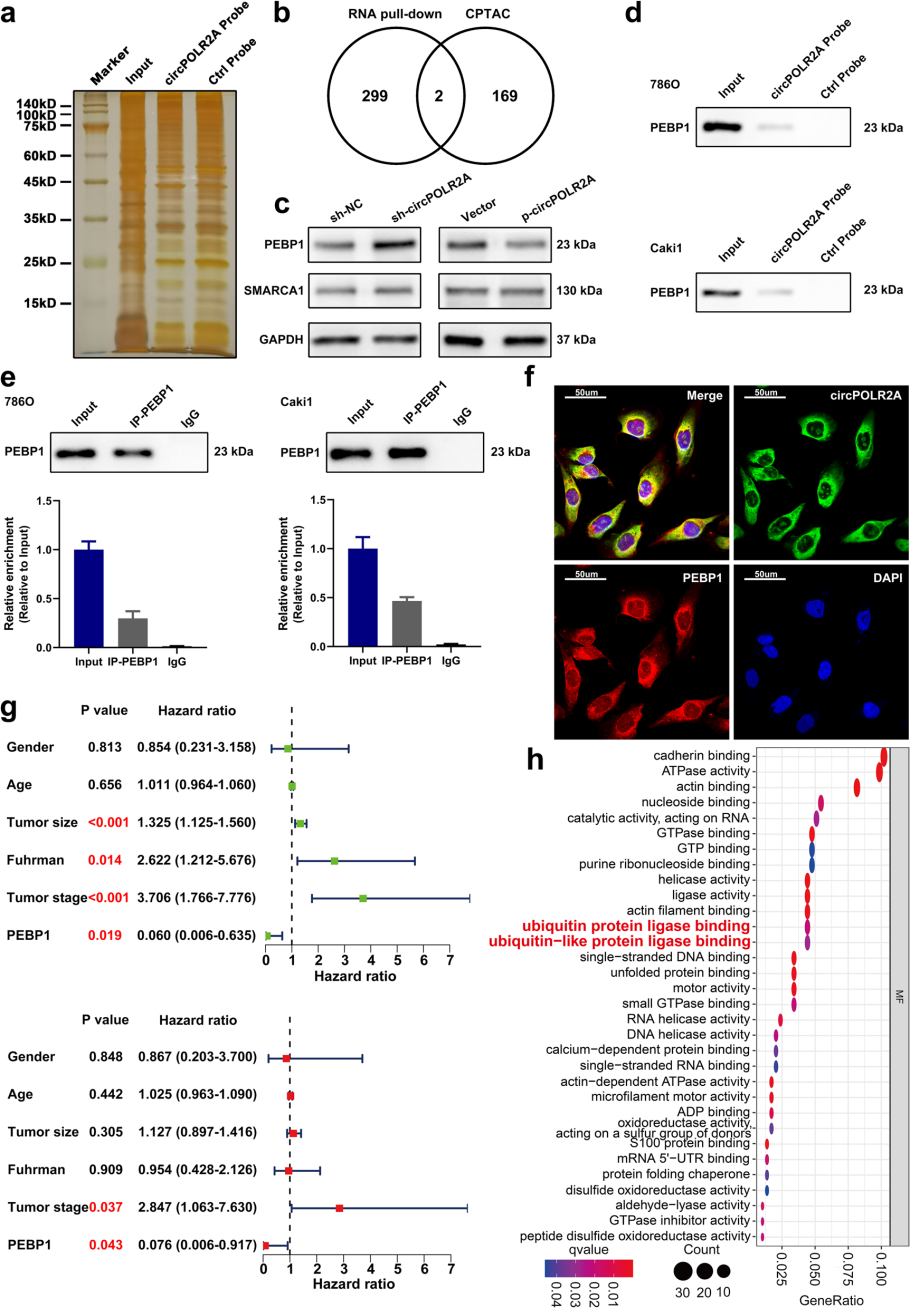
CircPOLR2A could interact with the PEBP1 protein. **a** RNA pull-down assay was performed in cRCC cells using a circPOLR2A sense probe (circPOLR2A probe) and an antisense probe (ctrl probe). **b** Venn diagram of the overlapping proteins (including PEBP1 and SMARCA1) between circPOLR2A-interacting proteins identified by MS and proteins with independent prognostic value in the cRCC cohort from the CPTAC database. **c** The effect of circPOLR2A on the protein levels of PEBP1 and SMARCA1 was assessed by western blotting. **d** RNA pull-down assay and western blotting validated the existence of PEBP1 in the precipitates pulled down with the circPOLR2A probe. **e** RIP assay validated the interaction between PEBP1 and circPOLR2A. Top, western blot analysis for IP efficiency of PEBP1 antibody; bottom, the enrichment of circPOLR2A in the precipitates of PEBP1 antibody relative to input group. **f** RNA FISH and immunofluorescence analysis indicated the colocalization of PEBP1 (red) and circPOLR2A (green) in 786O cells. **g** Forest plots showing the prognostic value of PEBP1 protein in the cRCC cohort from the CPTAC database. Top, the results of univariate Cox regression analysis (green dots); bottom, the results of multivariate Cox regression analysis (red dots). **h** GO enrichment analysis of circPOLR2A-interacting proteins identified via RNA pull-down and MS.

Based on previous findings, we further studied the potential mechanism by which circPOLR2A functioned as an oncogene in cRCC. First, we performed the pull-down assay in 786O cells using the biotin-labeled probe of circPOLR2A. As shown in Supplemental Fig. 2a, AGO2, a key protein participating in miRNA sponges, was not determined in the precipitates of the circPOLR2A probe via western blotting, which suggested that circPOLR2A did not act as a miRNA sponge in cRCC cells. The precipitates generated from the pull-down assay were detected via SDS-PAGE and silver staining (Fig. 4a). Subsequently, mass spectrometry (MS) analysis was used to determine the proteins interacting with circPOLR2A. The results indicated that 301 proteins were specifically detected in the precipitates of the circPOLR2A probe, and AGO2 was not among them. To further select circPOLR2A-related proteins with potential functions in cRCC, we conducted additional bioinformatics analysis. We performed integrated proteome analysis based on the National Cancer Institute’s Clinical Proteomic Tumor Analysis Consortium (CPTAC) database via R software, and the results indicated that 171 proteins could serve as independent prognostic factors in cRCC (Supplemental Table 3). Venn diagram showed that PEBP1 and SMARCA1 overlapped in circPOLR2A-interacting proteins and the proteins with independent prognostic value in cRCC (Fig. 4b). Western blotting was utilized to test whether the protein expression of PEBP1 and SMARCA1 was regulated by circPOLR2A knockdown or overexpression, and the results suggested that PEBP1 expression was negatively regulated by circPOLR2A, whereas no change in SMARCA1 expression was observed (Fig. 4c). In addition, qRT-PCR was performed to confirm that circPOLR2A knockdown or overexpression had no effect on the mRNA expression of PEBP1 (Supplemental Fig. 2b).

**Fig. 5:**
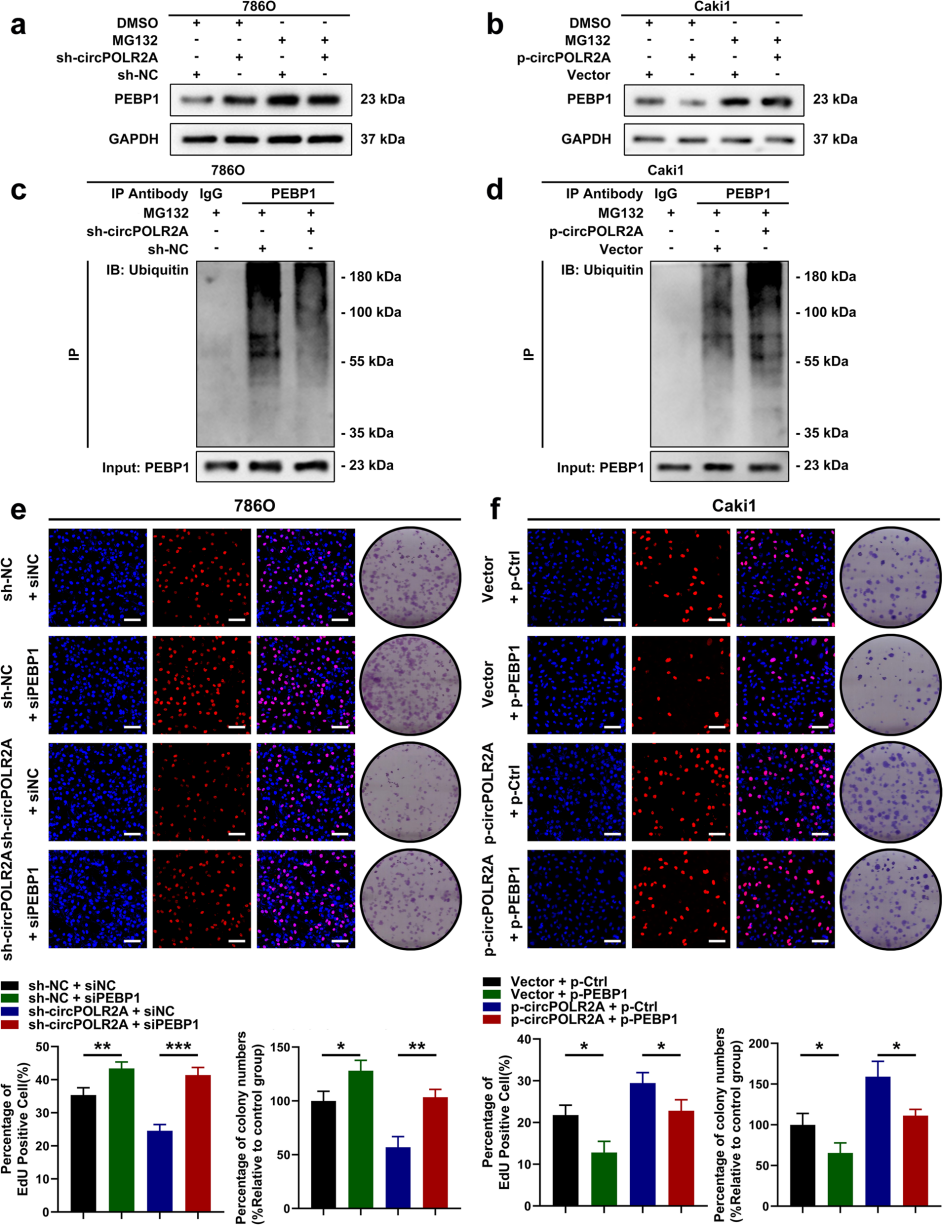
CircPOLR2A accelerated ubiquitin/proteasome-dependent protein degradation of PEBP1. **a**, **b** Protein level of PEBP1 in cRCC cells treated with 20 μM MG132 for 12 h. 5a, 786O cells transfected with circPOLR2A shRNA and control; 5b, Caki1 cells transfected with lentivirus for circPOLR2A overexpression and control. **c**, **d** Ubiquitination modification of PEBP1 proteins in cRCC cells was determined via Co-IP and western blotting. **e**, **f** EdU incorporation and colony formation assays were carried out for rescue experiments. 5e, the restoring effect of siPEBP1 on circPOLR2A downregulation in 786O cells; 5f, the restoring effect of ectopic PEBP1 expression on circPOLR2A upregulation in Caki1 cells. Scale bar, 100 μm

Considering the reported roles of PEBP1 reviewed in the introduction section and the data described above, we speculated that circPOLR2A could interact with PEBP1, which resulted in the downregulation of PEBP1. Pull-down assay and western blotting further validated that PEBP1 existed in the precipitates of the circPOLR2A probe (Fig. 4d). RNA immunoprecipitation (RIP) assay with a PEBP1-specific antibody confirmed that PEBP1 could recruit circPOLR2A (Fig. 4e). In addition, RNA FISH and immunofluorescence analysis suggested that circPOLR2A was colocalized with PEBP1 in the cytoplasm (Fig. 4f). Bioinformatics analysis indicated that the protein level of PEBP1 was negatively correlated with tumor size, pathologic T stage and Fuhrman grade in the cRCC cohort from the CPTAC database (Supplemental Fig. 2c, d and e). The results of univariate and multivariate Cox regression analyses and Kaplan-Meier curve analysis were displayed in Fig. 4g and Supplemental Fig. 2f, respectively, which suggested that the protein expression of PEBP1 was an independent prognostic factor for favorable prognosis in cRCC. In addition, we performed GO enrichment analysis on the results of mass spectrometry analysis (Supplemental Table 4). Intriguingly, circPOLR2A-related proteins were significantly enriched in ‘ubiquitin protein ligase binding’ and ‘ubiquitin-like protein ligase binding’ (Fig. 4h). In mammalian cells, the ubiquitin-proteasome system is the main cellular system with protease activity that mediates protein degradation [44–46]. Ubiquitylation starts with ubiquitin-activating enzymes (E1) and ubiquitin-conjugating enzymes (E2) which prepare ubiquitin for conjugation [47]. Then, ubiquitin ligases (E3) tag ubiquitin to the proteins, which conveys ubiquitin substrate specificity [47]. Finally, the selective proteins with ubiquitin tags are degraded by the 26S proteasome [48]. The GO enrichment analysis suggested that the effect of circPOLR2A on the protein expression of PEBP1 could be associated with protein ubiquitination and degradation pathways. Based on this assumption, we treated cRCC cells with MG132, an inhibitor of the proteasome, and the results revealed that the upregulation of PEBP1 protein mediated by circPOLR2A knockdown could be counteracted by MG132 (Fig. 5a). As shown in Fig. 5b, circPOLR2A overexpression inhibited the protein level of PEBP1 in Caki1 cells, which was restored by MG132. We further determined the ubiquitination level of PEBP1 under different conditions. Coimmunoprecipitation (Co-IP) and western blotting revealed that circPOLR2A knockdown significantly decreased the ubiquitination level of PEBP1 in 786O cells (Fig. 5c), while circPOLR2A overexpression promoted the ubiquitination of PEBP1 in Caki1 cells (Fig. 5d). Taken together, these data demonstrated that circPOLR2A inhibited the level of PEBP1 protein by ubiquitin/proteasome-dependent protein degradation in cRCC cells.

### PEBP1 was the functional downstream mediator of circPOLR2A

**Fig. 6:**
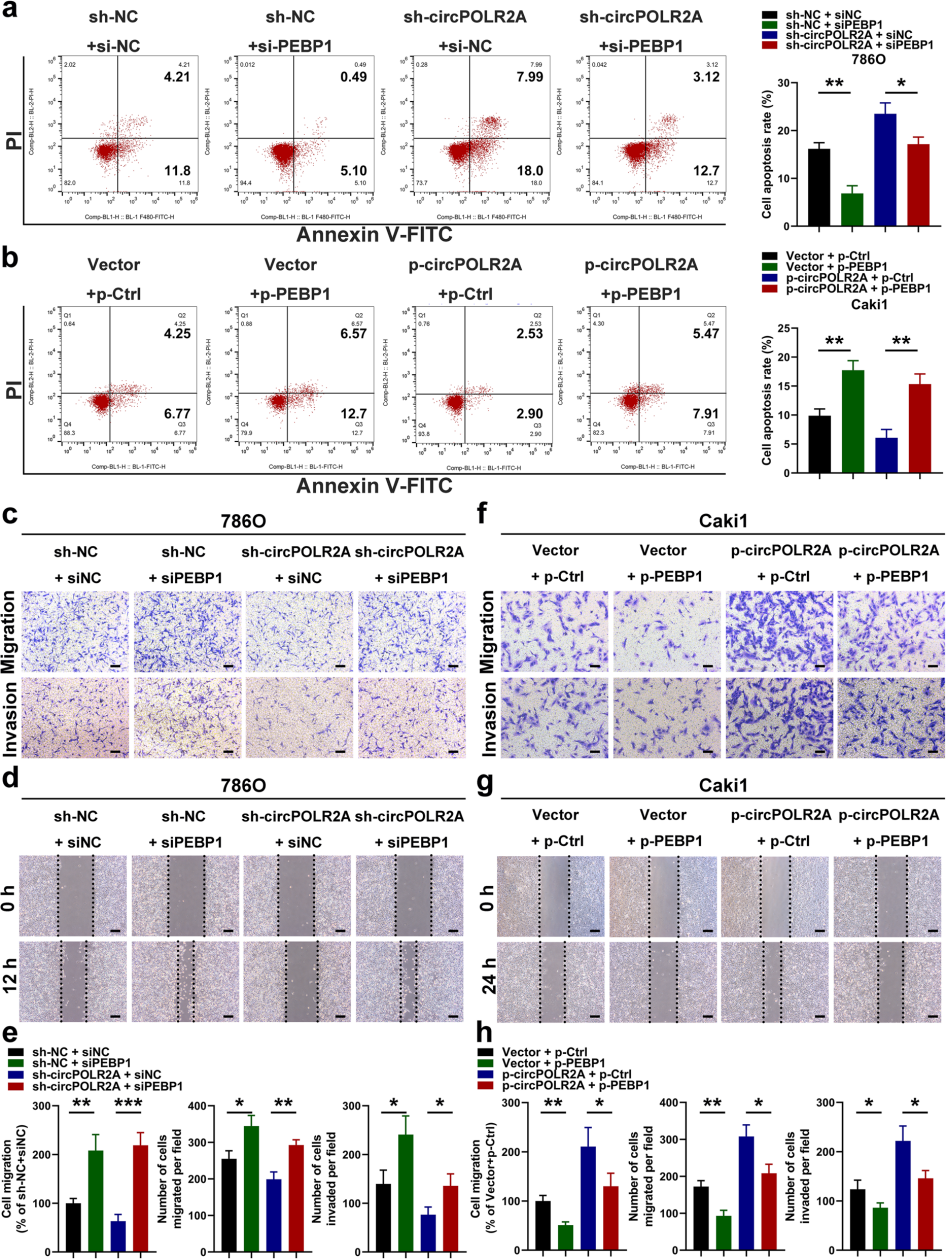
CircPOLR2A exerted its biological functions by inhibiting PEBP1 expression in cRCC cells. **a**, **b** Cell apoptosis was determined after cell transfection. 6a, 786O cells; 6b, Caki1 cells. **c**, **f** Transwell assay in rescue experiments. 6c, the effect of circPOLR2A knockdown in 786O cells could be counteracted by PEBP1 inhibition; 6f, the role of circPOLR2A overexpression in Caki1 cells could be rescued by PEBP1 overexpression. Scale bar, 40 μm. **d**, **g** Wound healing assay for rescue experiments. Scale bar, 100 μm. **e**, **h** Statistical analysis of the cell migration and invasion assays

To investigate whether circPOLR2A exerted its functions by regulating PEBP1 expression, we used siRNAs and plasmids for PEBP1 knockdown and overexpression in further experiments. As shown in Figs. 5e, 6a and c-e, we demonstrated that PEBP1 knockdown dramatically reversed the effect of circPOLR2A downregulation on cell proliferation, migration, invasion and apoptosis in 786O cells. Similarly, the positive effect of circPOLR2A overexpression on cell malignancy was counteracted by ectopic PEBP1 expression in Caki1 cells (Figs. 5f, 6b and f-h). These data suggested that circPOLR2A participated in cRCC cell malignancy by downregulating PEBP1 expression.

### UBE3C was a specific ubiquitin E3 ligase involved in PEBP1 ubiquitination

**Fig. 7:**
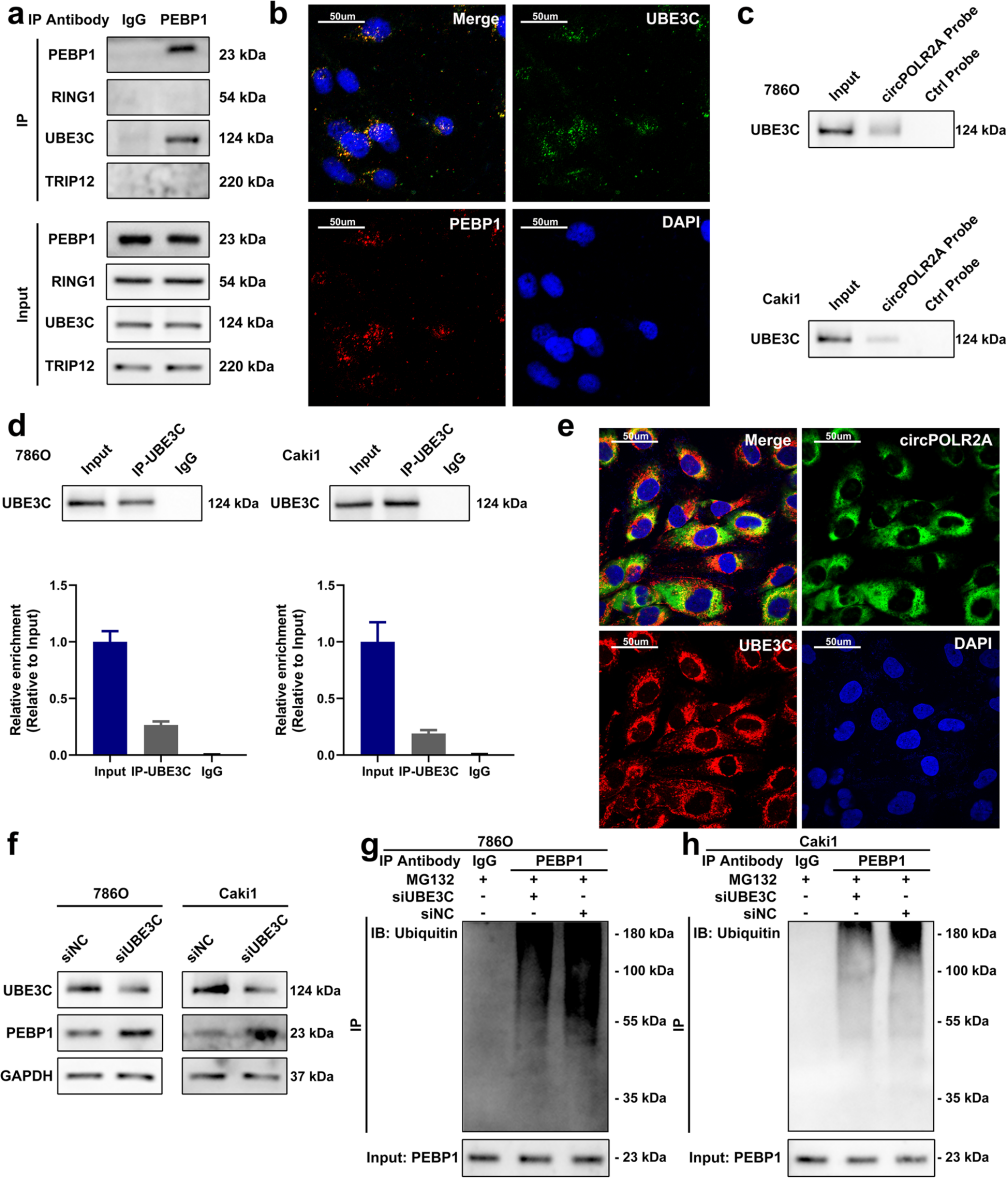
UBE3C was a ubiquitin E3 ligase which mediated the ubiquitination of PEBP1. **a** Co-IP with PEBP1 antibody confirmed the existence of UBE3C in the precipitates of PEBP1. **b** Immunofluorescence analysis of the localization of PEBP1 (red) and UBE3C (green). **c** RNA pull-down confirmed the binding of circPOLR2A and UBE3C. **d** RIP assay indicated the association between circPOLR2A and UBE3C. **e** RNA FISH and immunofluorescence analysis of the localization of UBE3C (red) and circPOLR2A (green). **f** The protein expression of PEBP1 after UBE3C knockdown in cRCC cells. Left, 786O cells; right, Caki1 cells. **g**, **h** PEBP1 ubiquitination was detected after UBE3C knockdown. 7 g, 786O cells; 7 h, Caki1 cells

We further explored the potential mechanism by which circPOLR2A mediated the ubiquitination and degradation of the PEBP1 protein in cRCC. It has been widely recognized that ubiquitin E3 ligases play an essential role in selecting specific substrates for ubiquitination reactions, which functions via the binding of ubiquitin E3 ligases and substrates [45]. Hence, we assumed that circPOLR2A could influence the interaction between ubiquitin E3 ligases and substrates, which resulted in the effect of circPOLR2A on PEBP1 ubiquitination. After a literature search, we found that no study reported the ubiquitination of PEBP1 and related ubiquitin E3 ligases. Next, we concentrated on the 3 ubiquitin E3 ligases (RING1, UBE3C and TRIP12) among the 301 proteins that interacted with circPOLR2A. Co-IP and western blotting were performed to identify which ubiquitin E3 ligase could specifically bind to the PEBP1 protein, and the results indicated that UBE3C could directly bind to the PEBP1 protein (Fig. 7a). Immunofluorescence analysis verified the colocalization of the PEBP1 protein and UBE3C protein (Fig. 7b). RNA pull-down and RIP assays confirmed the association between UBE3C and circPOLR2A (Fig. 7c, pull-down assay; 7d, RIP assay). RNA FISH and immunofluorescence analysis verified that UBE3C was colocalized with circPOLR2A in the cytoplasm (Fig. 7e). These results suggested that the UBE3C protein could interact with the PEBP1 protein and circPOLR2A.

Several studies have reported that UBE3C acts as a ubiquitin E3 ligase and participates in the ubiquitination of key molecules in cancers [49–52]. However, the potential effect of UBE3C on the PEBP1 protein remained unknown. Interestingly, the protein level of PEBP1 was significantly increased by UBE3C knockdown in cRCC cells (Fig. 7f). Furthermore, the ubiquitination level was dramatically suppressed by UBE3C knockdown (Fig. 7g and h). Taken together, these results indicated that UBE3C could serve as a ubiquitin E3 ligase which contributed to the degradation of PEBP1 protein through the ubiquitin-proteasome pathway in cRCC cells.

### circPOLR2A strengthened the interaction between UBE3C and PEBP1 in cRCC cells

**Fig. 8:**
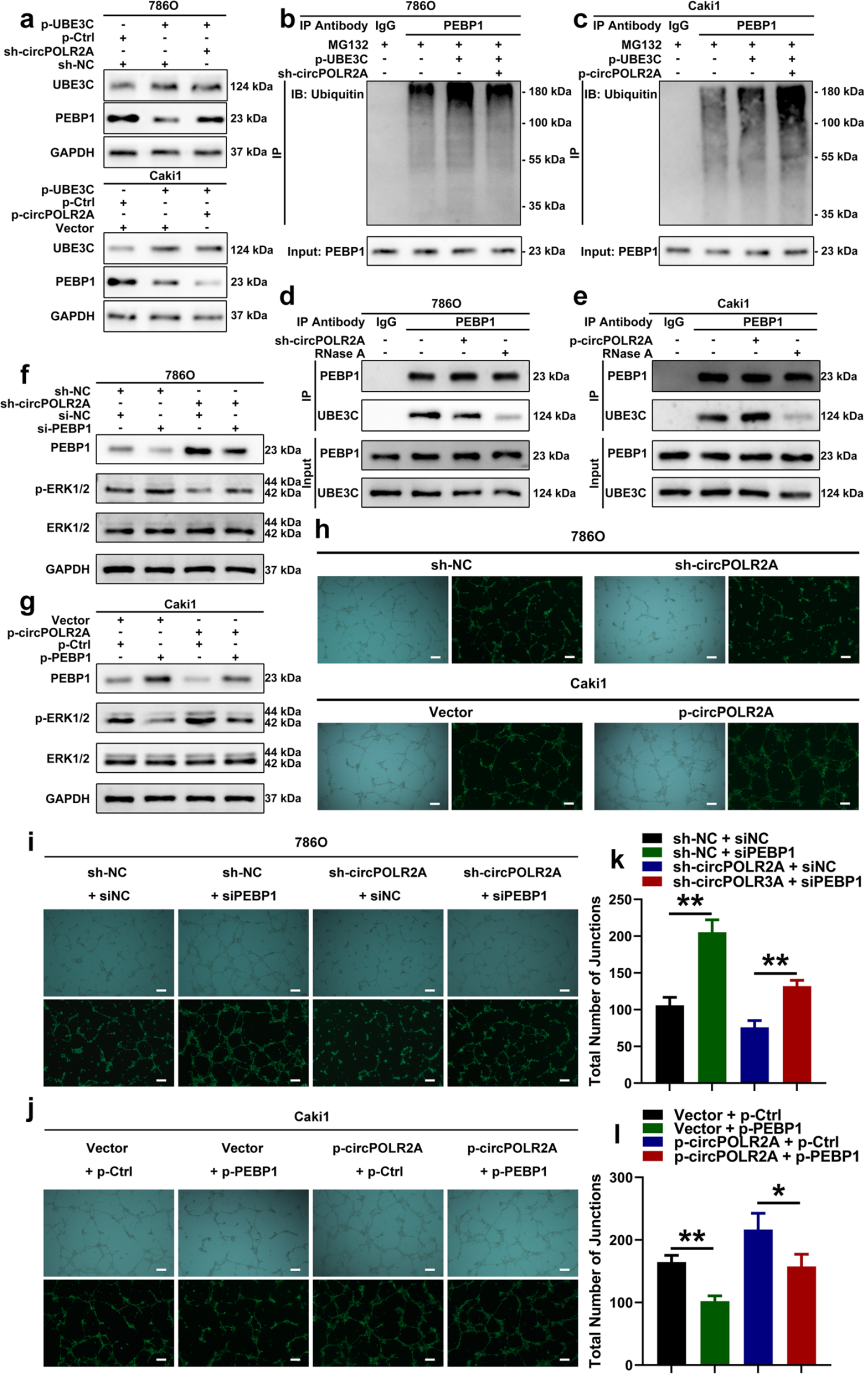
CircPOLR2A enhanced the UBE3C-mediated ubiquitination of PEBP1. **a** After circPOLR2A knockdown or overexpression, western blotting detected the PEBP1 protein level in cRCC cells with UBE3C overexpression. Top, 786O cells with circPOLR2A knockdown; bottom, Caki1 cells with ectopic circPOLR2A expression. **b**, **c** Ubiquitination modification of PEBP1 in cRCC cells after ectopic UBE3C expression and circPOLR2A knockdown or overexpression detected via Co-IP and western blotting. 8b, 786O cells with circPOLR2A knockdown; 8c, Caki1 cells with ectopic circPOLR2A expression. **d**, **e** The binding of PEBP1 and UBE3C in cRCC cells after transfection with circPOLR2A knockdown (8d) or overexpression (8e) and treatment with 10 μg/ml RNase A. **f**, **g** Western blotting verified the activation of the ERK pathway in cRCC cells. 8f, 786O cells; 8 g, Caki1 cells. **h** Tube formation assay evaluated the angiogenesis of HUVECs incubated with the culture medium from cRCC cells transfected with circPOLR2A knockdown or overexpression. Top, 786O cells; bottom, Caki1 cells. Scale bar, 100 μm. **i**, **j** Tube formation assay for rescue experiments. 8i, 786O cells; 8j, Caki1 cells. Scale bar, 100 μm. **k**, **l** Statistical analysis on total number of junctions in the tube formation assay for rescue experiments

Some researchers have reported that some RNAs can act as the scaffolds for protein-protein interactions [20, 53, 54]. Hence, we hypothesized that the existence of circPOLR2A was involved in the ubiquitin E3 ligase activity of UBE3C for PEBP1. As presented in Fig. 8a, the overexpression of UBE3C significantly decreased the protein level of PEBP1 in cRCC cells, which could be reversed by circPOLR2A knockdown and enhanced by ectopic circPOLR2A expression. Co-IP assay and western blotting suggested that the ubiquitination of PEBP1 induced by UBE3C overexpression was notably weakened by circPOLR2A knockdown (Fig. 8b), and further enhanced by circPOLR2A overexpression (Fig. 8c). To further verify the hypothesis, Co-IP analysis was performed. Intriguingly, knockdown of circPOLR2A weakened the interaction between UBE3C and PEBP1 (Fig. 8d), and ectopic circPOLR2A expression enhanced the interaction (Fig. 8e). However, the interaction was remarkably inhibited by RNase A, an RNA endonuclease, due to the sensitivity of circRNAs to RNA endonuclease (Fig. 8d and e). These results indicated that circPOLR2A served as the scaffold between PEBP1 and UBE3C, which promoted their interaction and the ubiquitination of PEBP1 for degradation.

### circPOLR2A was involved in activation of the ERK pathway and angiogenesis in cRCC

As mentioned in the introduction section, PEBP1 is closely associated with the Raf1/MEK/ERK pathway. By western blotting, we detected the phosphorylation of ERK1/2 to evaluate the activation of the ERK pathway in cRCC cells. After PEBP1 downregulation, the inhibitory effect of circPOLR2A knockdown on ERK1/2 phosphorylation was restored (Fig. 8f). Conversely, the level of phosphorylated ERK1/2 was induced by circPOLR2A overexpression, which could be reversed by ectopic PEBP1 expression (Fig. 8g). These data demonstrated that circPOLR2A could downregulate the protein level of PEBP1, which activated the ERK signaling pathway.

It has been reported that the activation of the ERK pathway plays an important role in angiogenesis in cancers [55–57]. Therefore, we performed tube formation assays using HUVECs to test whether the circPOLR2A/PEBP1 axis affected angiogenesis. The culture supernatant collected from cRCC cells was incubated with HUVECs. As presented in Fig. 8h and Supplemental Fig. 3a-b, circPOLR2A knockdown significantly reduced the total number of junctions and total vessels length in tube formation assays, whereas circPOLR2A overexpression exerted the opposite effect. Subsequently, the rescue assay indicated that PEBP1 silencing partially reversed the effect of circPOLR2A knockdown on angiogenesis, while ectopic PEBP1 expression counteracted the positive effect of circPOLR2A overexpression (Fig. 8i-l and Supplemental Fig. 3c-d). Taken together, these results revealed that circPOLR2A could exert a positive effect on angiogenesis by inhibiting PEBP1 expression in cRCC.

### circPOLR2A facilitated cRCC cell growth and metastasis in vivo

**Fig. 9:**
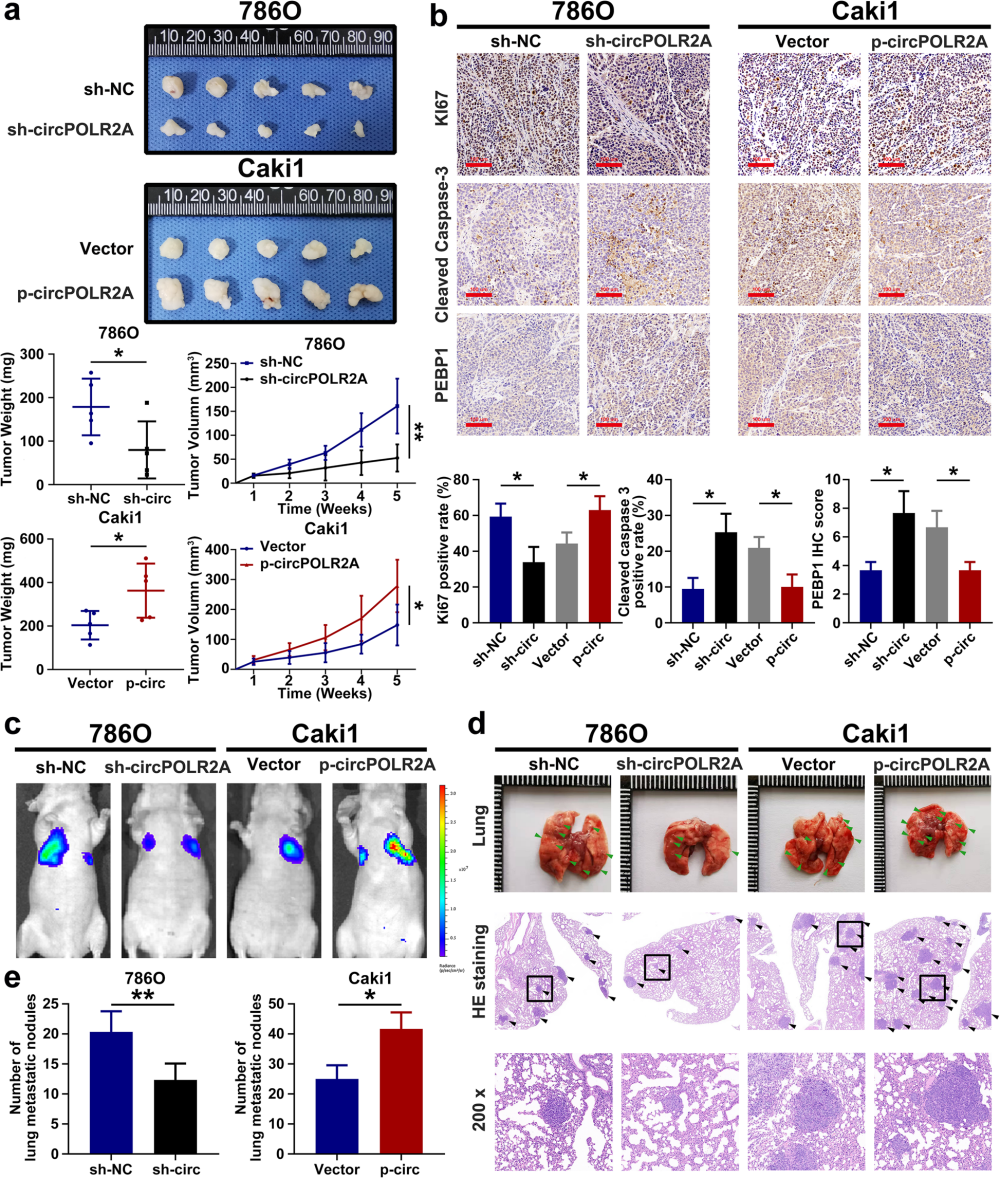
CircPOLR2A inhibited the growth and metastasis of cRCC cells in vivo. **a** Tumor size and weight of the subcutaneous tumor growth model injected with cRCC cells (5 mice/group); **b** IHC staining of the indicated proteins in the tumor samples obtained from the subcutaneous tumor growth model. Top, Ki-67; middle, cleaved caspase 3; bottom, PEBP1. **c** Representative bioluminescent images of lungs for each experimental group after 6 weeks (5 mice/group). **d** Representative images of lung and HE staining of lung metastases. **e** Statistical analysis of the number of metastatic nodules in lung

To investigate the in vivo effect of circPOLR2A on cRCC cell growth, cRCC cells were subcutaneously injected into nude mice. As shown in Fig. 9a, the cells with circPOLR2A knockdown showed markedly slower tumor growth and lower tumor weight in the xenograft mouse model, while circPOLR2A overexpression in cRCC cells led to the opposite effects. Immunohistochemistry (IHC) analysis was further performed to confirm the effect of circPOLR2A in vivo. The Ki67 distribution was significantly lower in tumors with circPOLR2A knockdown than in the control, and circPOLR2A overexpression displayed the opposite effects (Fig. 9b, top). Subsequently, cleaved caspase 3 staining verified that circPOLR2A could regulate cRCC cell apoptosis in vivo (Fig. 9b, middle). Furthermore, IHC analysis verified that PEBP1 protein expression was significantly reduced by circPOLR2A overexpression, whereas the opposite trend was demonstrated after circPOLR2A knockdown (Fig. 9b, bottom).

The metastatic models were constructed by tail vein injection of cRCC cells into nude mice. As shown in Fig. 9c, circPOLR2A overexpression promoted the metastasis of cRCC cells, whereas circPOLR2A knockdown suppressed the metastasis of cRCC cells. The HE staining verified the lung metastases (Fig. 9d). The incidence of lung metastases was inhibited by circPOLR2A knockdown, while ectopic circPOLR2A expression presented the opposite effects (Fig. 9e). Taken together, these data indicated that circPOLR2A could effectively promote tumor growth and metastasis in vivo.

### YTHDF2 played an inhibitory role on the expression of circPOLR2A, which was related to m6A modification

**Fig. 10:**
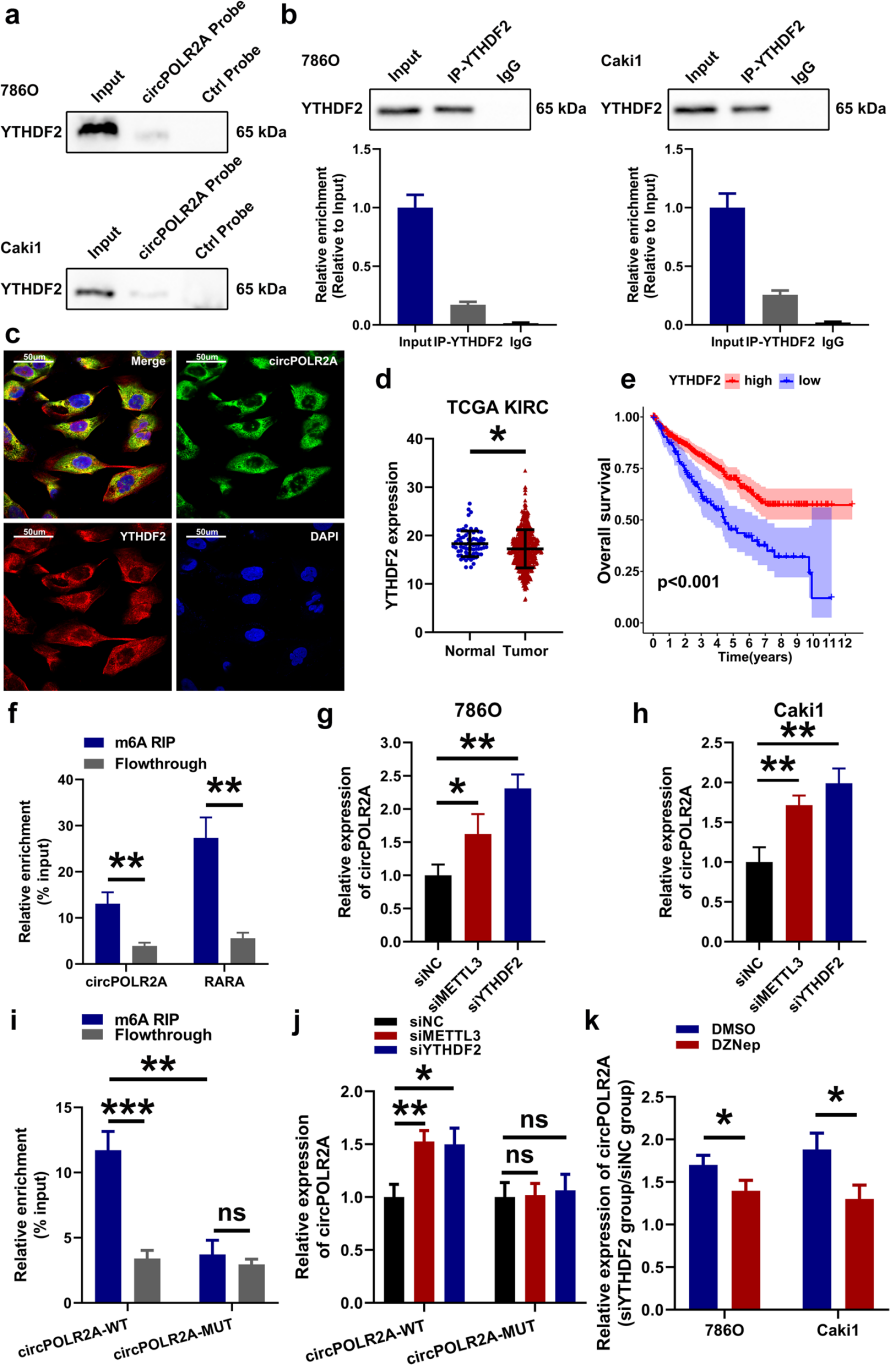
Knockdown of the m6A reader YTHDF2 promoted the expression of circPOLR2A in cRCC cells. **a** Pull-down assay validated the binding of YTHDF2 and circPOLR2A. Top, 786O cells; bottom, Caki1 cells. **b** RIP assay confirmed the binding of YTHDF2 and circPOLR2A. Left, 786O cells; right, Caki1 cells. **c** RNA FISH and immunofluorescence analysis of the colocalization of YTHDF2 (red) and circPOLR2A (green). **d** YTHDF2 expression was lower in cRCC tissues than in normal tissues based on TCGA database. **e** Kaplan-Meier method and log-rank test indicated that YTHDF2 expression was a favorable prognostic factor in the TCGA-KIRC cohort. **f** MeRIP assay verified the enrichment of circPOLR2A in m6A-precipitated fraction. **g**-**h** The expression of circPOLR2A in cRCC cells transfected with siNC, siMETTL3 or siYTHDF2. 10 g, 786O; 10 h, Caki1. **i** MeRIP assay determined the relative enrichment of circPOLR2A-WT or circPOLR2A-MUT in m6A-precipitated fraction in 786O cells transfected with circPOLR2A-WT or circPOLR2A-MUT plasmids. **j** Analysis of the relative abundance of circPOLR2A-WT or circPOLR2A-MUT in circPOLR2A-WT-expressing or circPOLR2A-MUT-expressing 786O cells transfected with siNC, siMETTL3 or siYTHDF2. **k** Analysis on the relative abundance of circPOLR2A in cRCC cells transfected with siNC or siYTHDF2. Blue, cRCC cells were pretreated with DMSO; Red, cRCC cells were pretreated with 10 μM DZNep for 1 h

Based on the results of MS analysis, we found that the m6A reader YTHDF2 was one of the potential circPOLR2A-interacting proteins. Considering the reported functions of YTHDF2 on RNA metabolism [41], it was assumed that YTHDF2 participated in the RNA metabolism of circPOLR2A. First, RNA pull-down (Fig. 10a) and RIP assays (Fig. 10b) further validated that circPOLR2A could bind with YTHDF2 protein. FISH and immunofluorescence analysis confirmed that YTHDF2 was colocalized with circPOLR2A (Fig. 10c). Next, western blotting indicated that circPOLR2A knockdown or overexpression exerted no significant effect on YTHDF2 expression (Supplemental Fig. 4c). In addition, we analyzed the expression and prognostic value of YTHDF2 based on the TCGA database. In the TCGA-KIRC cohort, YTHDF2 was downregulated in cRCC samples compared to normal samples (Fig. 10d). The cRCC patients with G3 or G4 grade showed significantly lower YTHDF2 expression than patients with G1 or G2 grade (Supplemental Fig. 4a). Similarly, lower YTHDF2 expression was determined in cRCC patients with stage III or IV than in patients with stage I or II (Supplemental Fig. 4b). Furthermore, patients with higher expression of YTHDF2 have a favorable prognosis in the cRCC cohort (Fig. 10e).

Since YTHDF2 functioned on RNA metabolism via the m6A-dependent manner, we identified putative m6A motifs by browsing the sequence of circPOLR2A in SRAMP, a sequence-based m6A modification site predictor [58]. As shown in Supplemental Fig. 4d, there were 4 putative m6A motifs with high or very high confidence in circPOLR2A sequence. Subsequently, methylated RNA immunoprecipitation (MeRIP) analysis indicated that the recognized m6A-modified RNA, RARA [59], was markedly precipitated, and circPOLR2A was also significantly enriched in the m6A-precipitated fraction (Fig. 10f). To explore the potential m6A-dependent function of YTHDF2 on circPOLR2A, we used siRNAs to knockdown the expression of METTL3, the m6A writer, and YTHDF2, the m6A reader. Following transfection into cRCC cells, qRT-PCR was performed. The data suggested that knockdown of METTL3 or YTHDF2 was effective in cRCC cells (Supplemental Fig. 4e-h). As displayed in Fig. 10g and h, the expression of circPOLR2A was significantly induced by METTL3 or YTHDF2 knockdown in cRCC cells. We constructed a circPOLR2A plasmid with mutations in the m6A sites, namely, circPOLR2A-MUT (Supplemental Fig. 4i and j). 786O cells were transfected with the circPOLR2A-WT or circPOLR2A-MUT plasmids. MeRIP analysis indicated that circPOLR2A-WT exhibited higher m6A modification levels than circPOLR2A-MUT (Fig. 10i). As shown in Fig. 10j, higher circPOLR2A-WT expression was determined after METTL3 or YTHDF2 knockdown, while the abundance of circPOLR2A-MUT was not altered after METTL3 or YTHDF2 knockdown. In addition, we used 3-deazaneplanocin A (DZNep), an inhibitor of RNA methylation [60, 61], to further demonstrate whether the effect of YTHDF2 on circPOLR2A expression was associated with m6A modification. As shown in Fig. 10k, circPOLR2A expression induced by YTHDF2 knockdown was significantly weakened after DZNep treatment. Taken together, these results indicated that circPOLR2A could be modified by m6A, and that the m6A reader, YTHDF2, could regulate circPOLR2A expression in a m6A-dependent manner in cRCC cells.

## Discussion

**Fig. 11:**
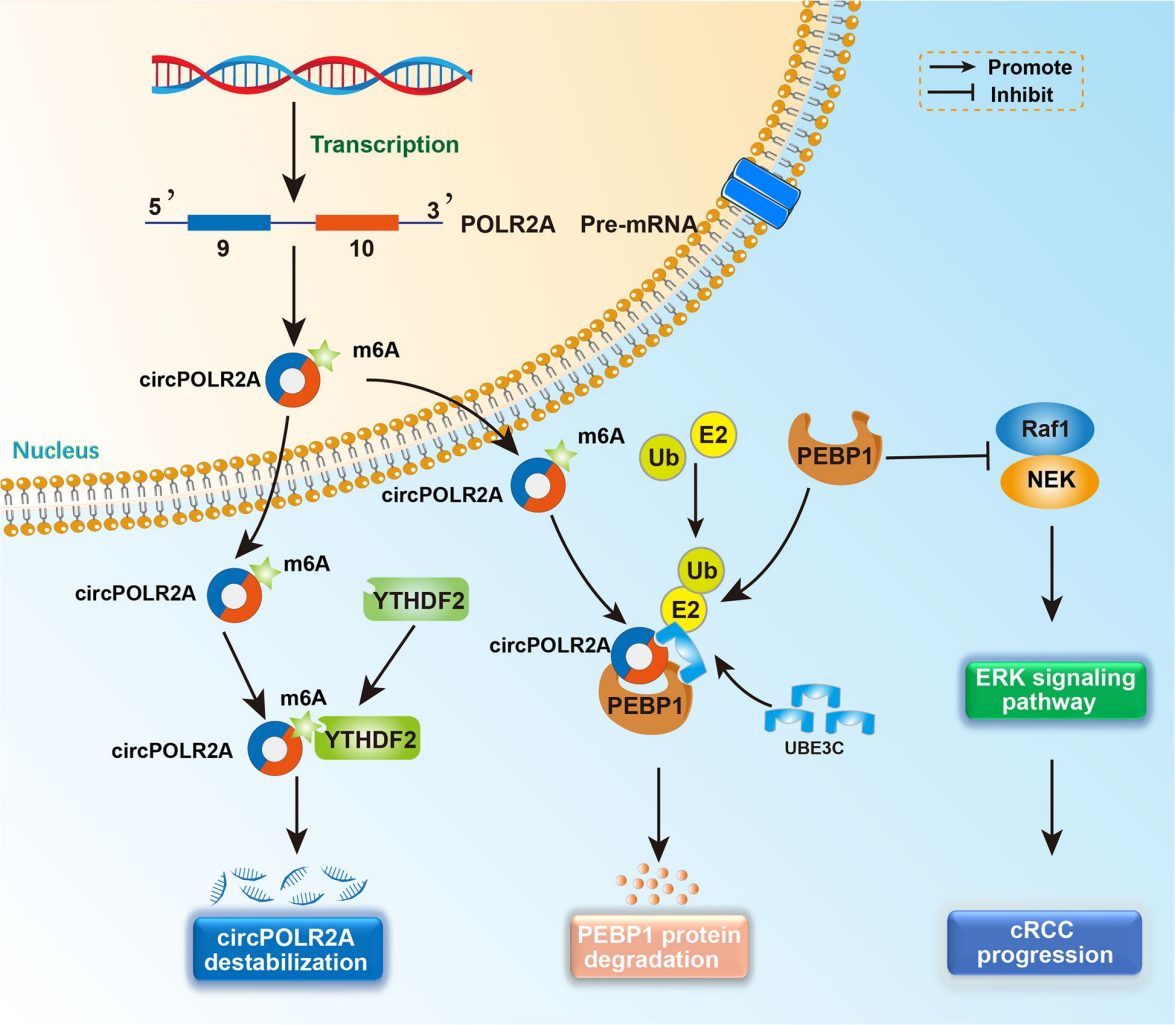
Proposed model for the potential function of circPOLR2A in cRCC progression. In the nucleus, circPOLR2A was derived from exons 9 and 10 of the POLR2A gene by backsplicing. After export to the cytoplasm, circPOLR2A interacted with UBE3C and PEBP1 proteins, and formed a circPOLR2A/UBE3C/PEBP1 protein-RNA ternary complex, which facilitated the ubiquitination and degradation of PEBP1 and further activated ERK signaling in cRCC progression. Furthermore, circPOLR2A could be modified by m6A, and the m6A reader YTHDF2 inhibited circPOLR2A expression in cRCC

Initially, circRNAs, arising from aberrant splicing events, were considered the ‘junk’ with little biological function [62–64]. Over the past decade, multiple circRNAs have been identified through advanced high-throughput RNA sequencing and specific bioinformatics algorithms [65–69]. Considering the conserved and stable features of circRNAs, an increasing number of researchers have concentrated on the biological functions of circRNAs in tumorigenesis and progression [12, 14]. Herein, we demonstrated for the first time that circPOLR2A was frequently highly expressed in cRCC tissues and cell lines. Increased expression of circPOLR2A induced the ubiquitination-mediated degradation of the PEBP1 protein by forming a circPOLR2A/UBE3C/PEBP1 protein-RNA ternary complex, which promoted cRCC malignancy (Fig. 11). In addition, we found that the metabolism of circPOLR2A could be modulated by YTHDF2 in a m6A-dependent manner in cRCC (Fig. 11).

In cRCC, most researchers have reported the functions of circRNAs as miRNA sponges [15–17, 70]. In our study, we validated that circPOLR2A acted as an oncogene not via the ceRNA mechanism, but through interacting with the PEBP1 and UBE3C proteins and further inhibiting the stability of the PEBP1 protein. It has been reported that PEBP1 could serve as a suppressor in cancer progression via the Raf1/MEK/ERK signaling pathway [71–74]. Nevertheless, the degradation of PEBP1 protein remained unknown. In this study, we validated the ubiquitination-mediated degradation of the PEBP1 protein in cRCC for the first time. We further identified UBE3C as a specific ubiquitin E3 ligase targeting the PEBP1 protein. In addition, the ubiquitin E3 ligase activity of UBE3C on PEBP1 protein could be strengthened by circPOLR2A in cRCC cells. Therefore, our study revealed a novel mechanism involved in the metabolism of PEBP1 protein.

With insights into m6A modification, the potential effects of m6A on circRNAs have attracted more attention [75–77]. Similar to mRNAs, m6A-modified circRNAs were eventually recognized by m6A readers [40, 78]. Specifically, Yang et al. claimed that YTHDF3, a m6A reader, was necessary for the m6A-driven translation of circRNAs [40]. Recently, YTHDC1 has been demonstrated to play an effective role in the translocation of circNSUN2 to the cytoplasm in a m6A-dependent manner [21]. Li et al. reported that the m6A readers, IGF2BPs, interacted with the m6A-modified circNDUFB2, which eventually modulated anti-tumor immunity in non-small-cell lung cancer [20]. Our study validated the interplay of circPOLR2A with the m6A reader, YTHDF2. Previous studies have revealed that YTHDF2 is able to destabilize m6A-modified RNA [79–81]. A novel study on hepatocellular carcinoma found that YTHDF2 facilitated the degradation of circCPSF6 containing m6A modification [41]. Coincidentally, our research demonstrated that YTHDF2 could regulate the expression of circPOLR2A in cRCC cells, which was related to m6A modification of RNA. Our study provides new evidence that m6A modification participates in the metabolism of circRNAs.

Our work indicated that circPOLR2A functioned in cRCC by enhancing the ubiquitination degradation of PEBP1 mediated by the ubiquitin E3 ligase, UBE3C, and revealed crosstalk between circPOLR2A and the m6A reader, YTHDF2. However, some limitations existed in our study. First, our study did not include enough clinical samples, and there was a lack of relevant follow-up prognostic information, which made it difficult for us to analyze the correlation between circPOLR2A expression and cRCC prognosis. Due to the stability of circRNAs endowed by their loop structure, several studies have demonstrated the clinical application potential of circRNAs as cancer biomarkers [77, 82–84]. Hence, including more cRCC samples with sufficient follow-up data is necessary for evaluating the clinical value of circPOLR2A in future research, which is of great significance for subsequent clinical transformation. Second, although we revealed a partial association between circPOLR2A and m6A modification, whether the generation process of circPOLR2A is regulated by m6A modification or other factors still requires further investigation.

## Conclusion

Our study demonstrates that circPOLR2A acts as an oncogene in cRCC, which promotes cell proliferation, migration, invasion and angiogenesis, and inhibits cell apoptosis. CircPOLR2A modulates the ERK signaling pathway by forming a circPOLR2A/UBE3C/PEBP1 protein-RNA ternary complex and further enhancing the UBE3C-mediated ubiquitination degradation of the PEBP1 protein. CircPOLR2A expression can be regulated by the m6A reader, YTHDF2, in cRCC. Our study broadens the knowledge of circRNAs in cRCC, which may provide a potential biomarker and therapeutic target for the diagnosis and treatment of human cRCC.

## Supplementary Information

**Additional file 1: Supplemental Fig. 1.** The expression of the 9 candidate circRNAs except circPOLR2A in 32 paired tissues of cRCC. *P* values were evaluated by paired two-sided t test.

**Additional file 2: Supplemental Fig. 2.** (a) Western blot indicated that no AGO2 protein was detected in the precipitates of RNA pull-down. (b) No significant change in PEBP1 mRNA expression was determined after circPOLR2A knockdown or overexpression. (c, d, e) Bioinformatics analysis of the CPTAC database suggested that PEBP1 protein levels were associated with cRCC tumor size (c), pathologic T stage (d) and Fuhrman grade (e) in the cRCC cohort. (f) Kaplan-Meier method and log-rank test confirmed that the PEBP1 protein level was a favorable prognostic factor in the cRCC cohort from CPTAC database.

**Additional file 3: Supplemental Fig. 3.** Statistical analysis on the tube formation assay. (a) Total number of junctions in the tube formation assay for circPOLR2A knockdown or overexpression. (b) Total vessels length in the tube formation assay for circPOLR2A knockdown or overexpression. (c, d) Total vessels length in the tube formation assay for rescue experiments.

**Additional file 4: Supplemental Fig. 4.** (a, b) Bioinformatics analysis suggested that YTHDF2 expression was associated with cRCC grade (a) and stage (b) in the cRCC cohort from TCGA database. (c) Western blotting detected the YTHDF2 level after circPOLR2A knockdown or overexpression. (d) The 4 putative m6A motifs of circPOLR2A predicted by the SRAMP prediction server. (e-h) The expression of METTL3 and YTHDF2 in cRCC cells transfected with siNC, siMETTL3 or siYTHDF2. 10e, 10f, METTL3 expression; 10 g, 10 h, YTHDF2 expression. (i, j) The schematic diagram illustrated the structure of circPOLR2A-WT (i) or circPOLR2A-MUT (j).

**Additional file 5: Supplemental Table 1.**

**Additional file 6: Supplemental Table 2.**

**Additional file 7: Supplemental Table 3.**

**Additional file 8: Supplemental Table 4.**

**Additional file 9: Supplemental Table 5.**

**Additional file 10: Supplemental Table 6.**

**Additional file 11. **Supplemental Methods and Materials**.**

## Data Availability

Please contact the corresponding author for data requests.

## Abbreviations

RCC: Renal cell carcinoma
cRCC: clear cell renal cell carcinoma
mRCC: metastatic renal cell carcinoma
circRNA: circular RNA
poly-(A) tail: polyadenylation tail
PEBP1: Phosphatidylethanolamine Binding Protein 1
RIP: RNA immunoprecipitation
Co-IP: Co-immunoprecipitation
m6A: N6-methyladenosine
YTHDF: YTH N6-Methyladenosine RNA Binding Protein
GEO: Gene Expression Omnibus
DE: Differentially expressed
qRT-PCR: quantitative reverse transcription PCR
gDNA: genomic DNA
cDNA: complementary DNA
FISH: Fluorescence in situ hybridization
MS: Mass spectrometry
CPTAC: Clinical Proteomic Tumor Analysis Consortium
IHC: Immunohistochemistry
MeRIP: Methylated RNA immunoprecipitation
TCGA: The Cancer Genome Atlas
