# Supplementary figures and images for: Circular RNA circPOLR2A promotes clear cell renal cell carcinoma progression by facilitating the UBE3C-induced ubiquitination of PEBP1 and, thereby, activating the ERK signaling pathway
Source: Mol Cancer. 2022 Jul 15;21:146. doi: 10.1186/s12943-022-01607-8 (PMC9284792; doi:10.1186/s12943-022-01607-8)

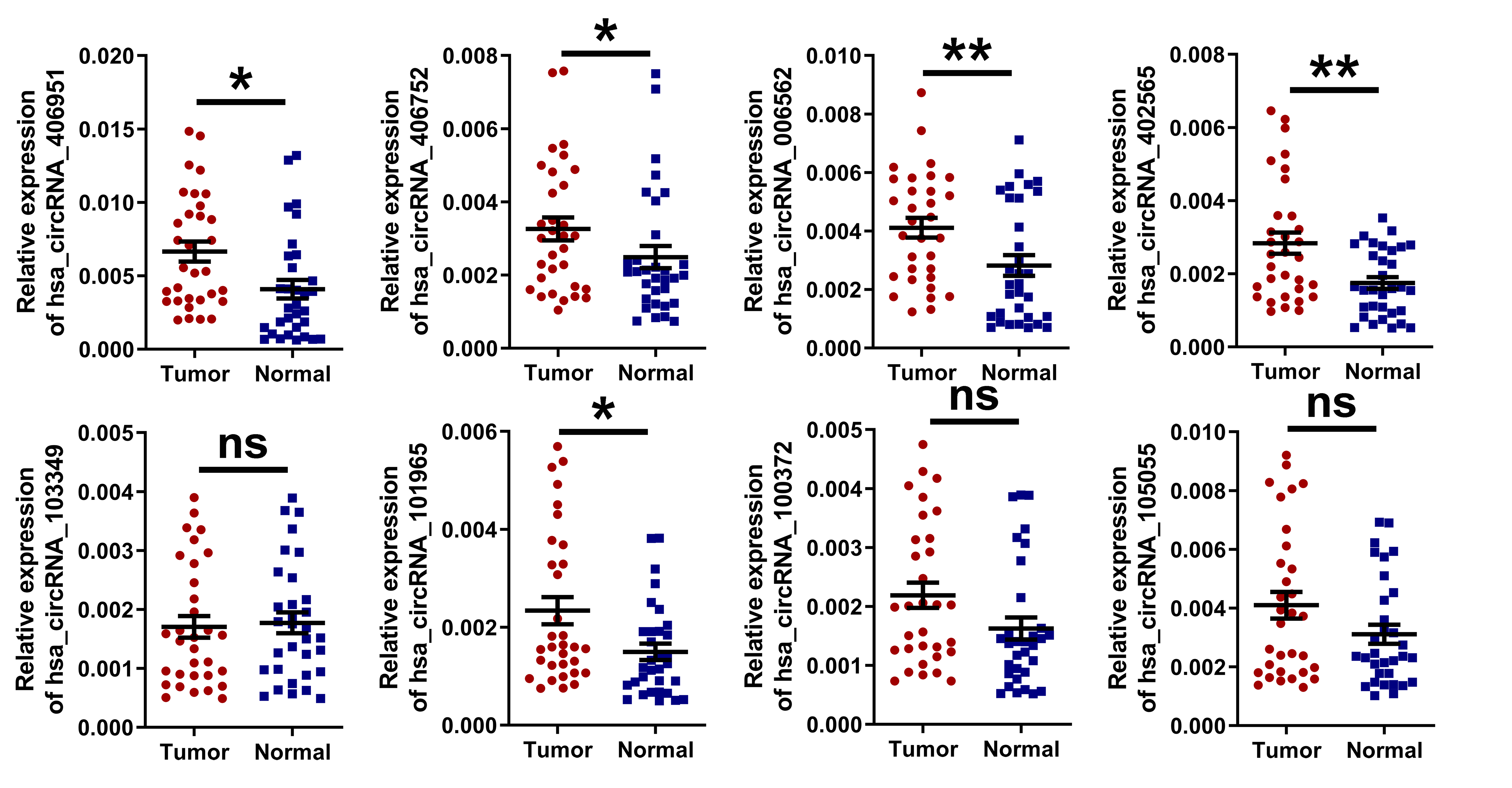

Supplement: Supplementary file 1 — Additional file 1: Supplemental Fig. 1. The expression of the 9 candidate circRNAs except circPOLR2A in 32 paired tissues of cRCC. P values were evaluated by paired two-sided t test. [file 12943_2022_1607_MOESM1_ESM.tif]

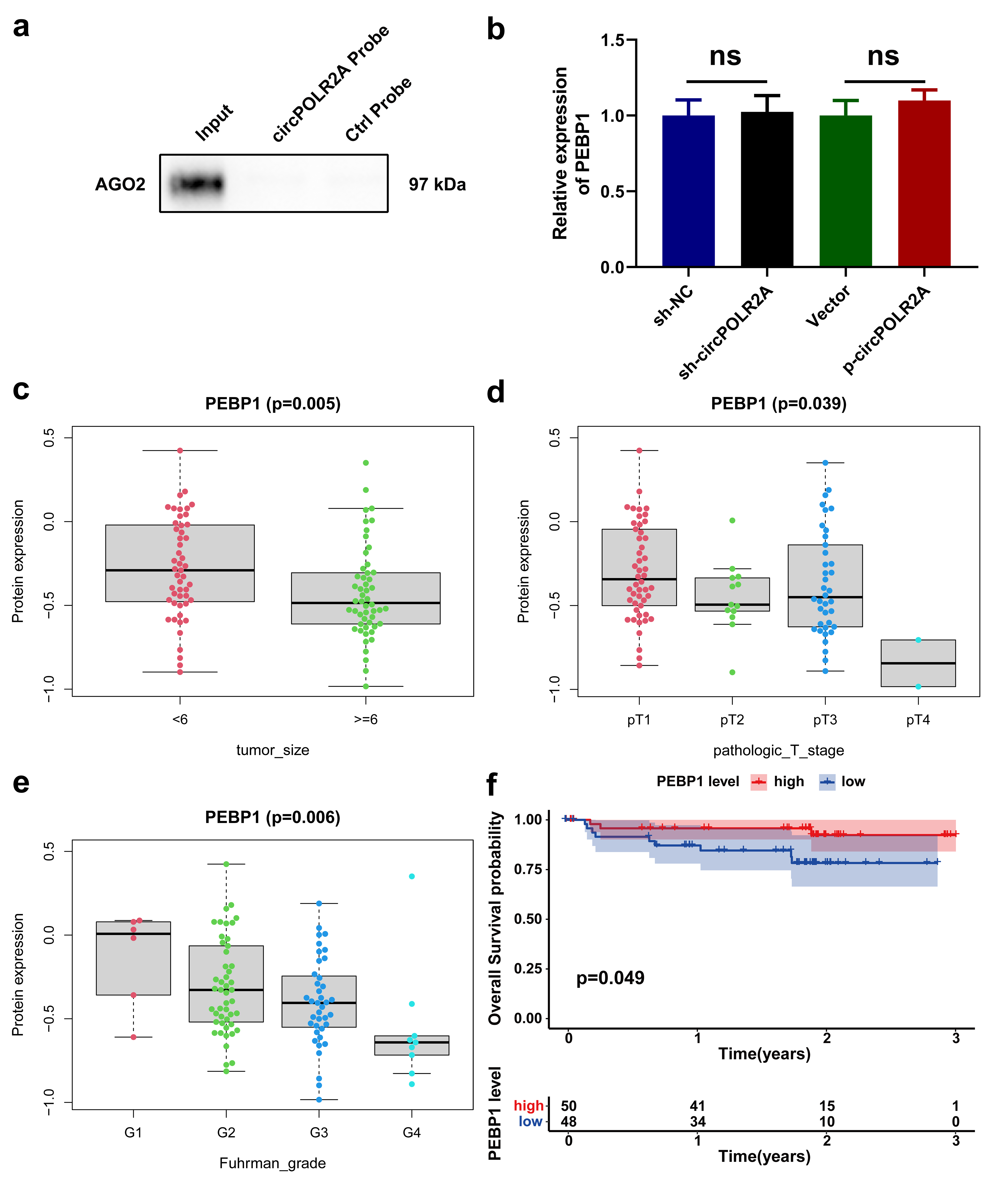

Supplement: Supplementary file 2 — Additional file 2: Supplemental Fig. 2. (a) Western blot indicated that no AGO2 protein was detected in the precipitates of RNA pull-down. (b) No significant change in PEBP1 mRNA expression was determined after circPOLR2A knockdown or overexpression. (c, d, e) Bioinformatics analysis of the CPTAC database suggested that PEBP1 protein levels were associated with cRCC tumor size (c), pathologic T stage (d) and Fuhrman grade (e) in the cRCC cohort. (f) Kaplan-Meier method and log-rank test confirmed that the PEBP1 protein level was a favorable prognostic factor in the cRCC cohort from CPTAC database. [file 12943_2022_1607_MOESM2_ESM.tif]

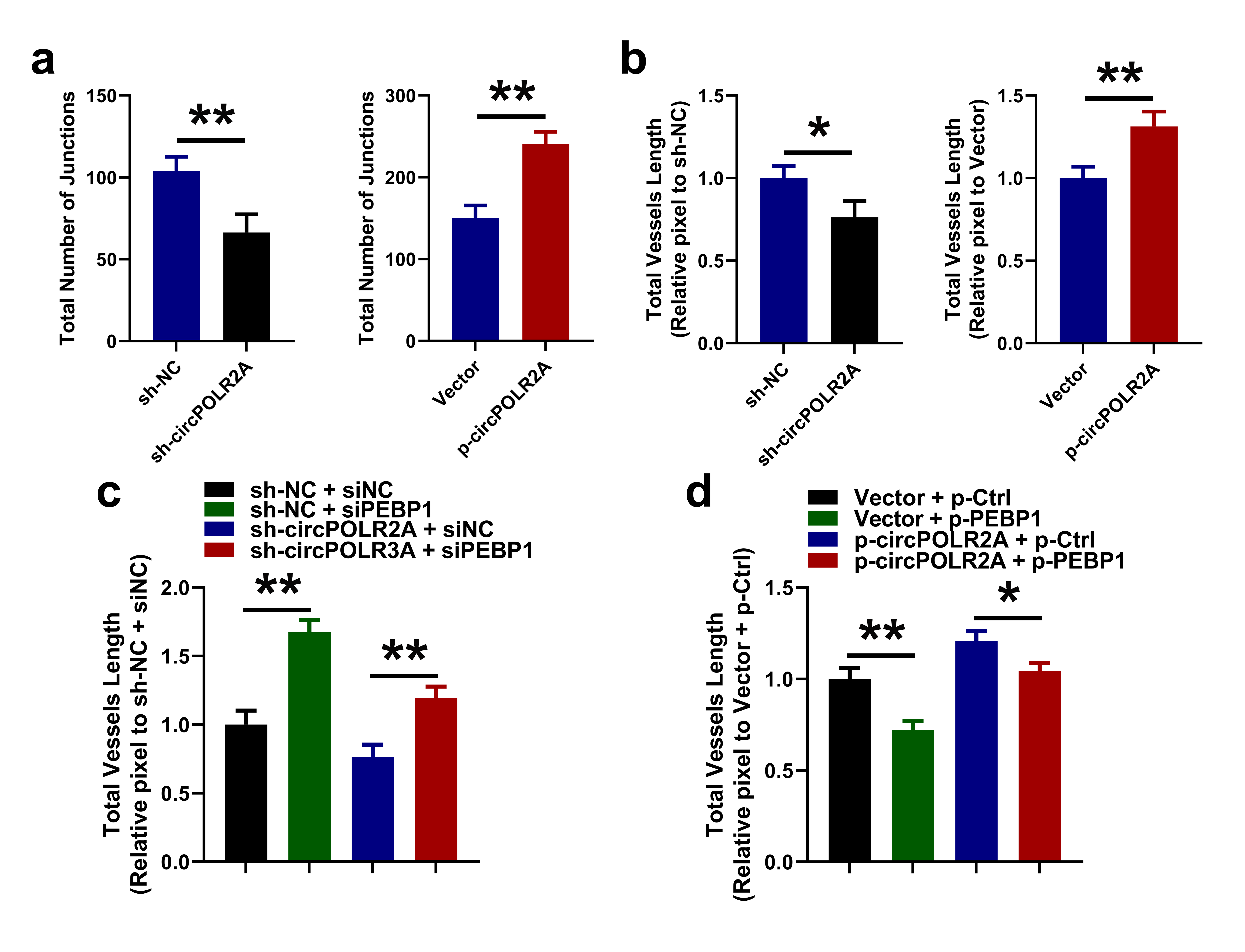

Supplement: Supplementary file 3 — Additional file 3: Supplemental Fig. 3. Statistical analysis on the tube formation assay. (a) Total number of junctions in the tube formation assay for circPOLR2A knockdown or overexpression. (b) Total vessels length in the tube formation assay for circPOLR2A knockdown or overexpression. (c, d) Total vessels length in the tube formation assay for rescue experiments. [file 12943_2022_1607_MOESM3_ESM.tif]

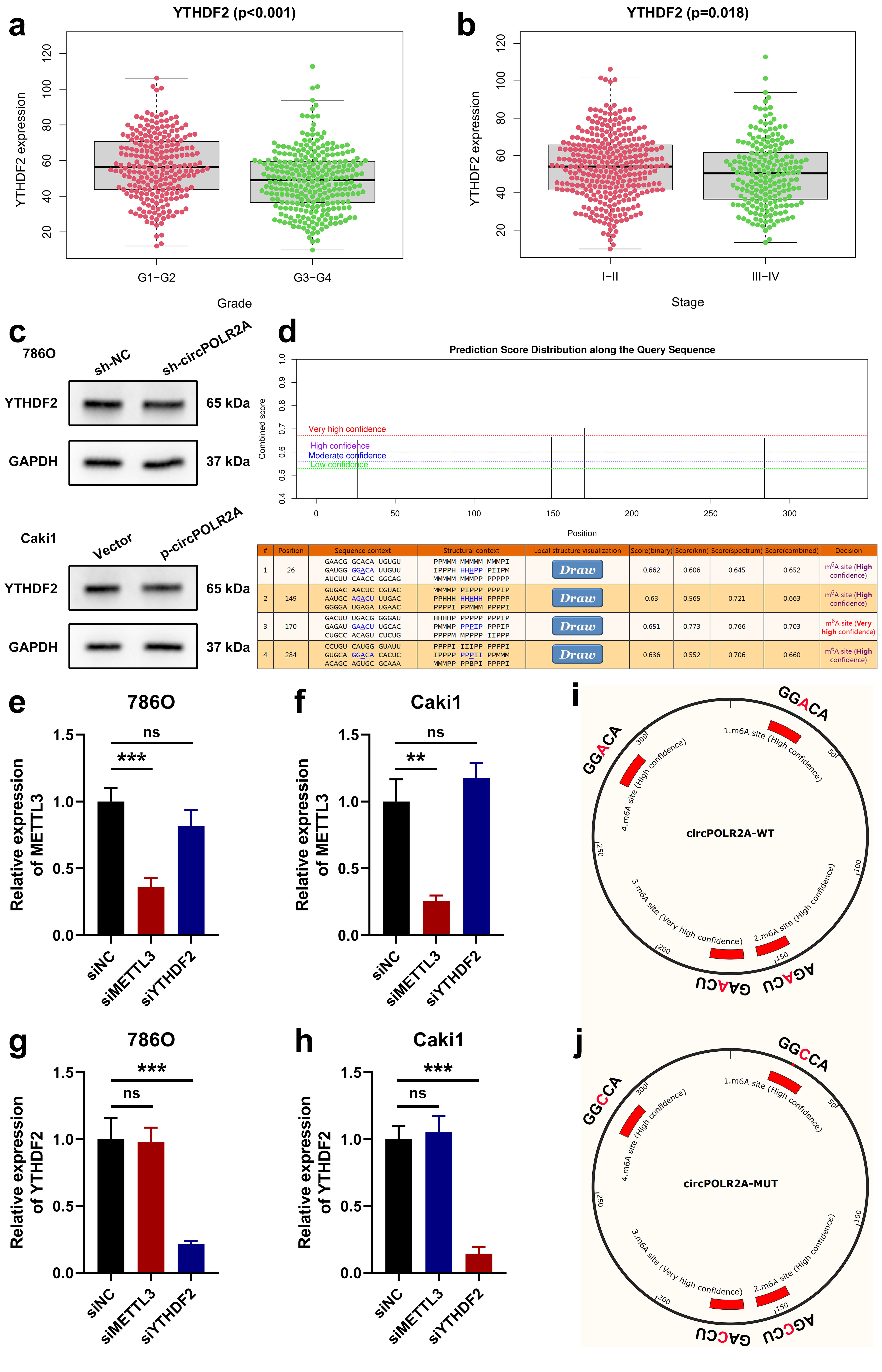

Supplement: Supplementary file 4 — Additional file 4: Supplemental Fig. 4. (a, b) Bioinformatics analysis suggested that YTHDF2 expression was associated with cRCC grade (a) and stage (b) in the cRCC cohort from TCGA database. (c) Western blotting detected the YTHDF2 level after circPOLR2A knockdown or overexpression. (d) The 4 putative m6A motifs of circPOLR2A predicted by the SRAMP prediction server. (e-h) The expression of METTL3 and YTHDF2 in cRCC cells transfected with siNC, siMETTL3 or siYTHDF2. 10e, 10f, METTL3 expression; 10 g, 10 h, YTHDF2 expression. (i, j) The schematic diagram illustrated the structure of circPOLR2A-WT (i) or circPOLR2A-MUT (j). [file 12943_2022_1607_MOESM4_ESM.tif]
