# Supplementary material for: Circular RNA circPOLR2A promotes clear cell renal cell carcinoma progression by facilitating the UBE3C-induced ubiquitination of PEBP1 and, thereby, activating the ERK signaling pathway
Source: Mol Cancer. 2022 Jul 15;21:146. doi: 10.1186/s12943-022-01607-8 (PMC9284792; doi:10.1186/s12943-022-01607-8)
Supplement: Supplementary file 5 — Additional file 5: Supplemental Table 1. [file 12943_2022_1607_MOESM5_ESM.docx]

**Supplemental table 1: The selected circRNA datasets on cRCC**

| **GEO accession** | **Author** | **Platform** | **Samples** | **Year** | **Tissue** |
| --- | --- | --- | --- | --- | --- |
| GSE137836 | Li W | GPL21825  074301 Arraystar Human CircRNA microarray V2 | 3：3 | 2019 | Primary tumor  Metastatic tumor |
| GSE100186 | Lv Q | GPL21825  074301 Arraystar Human CircRNA microarray V2 | 4：4 | 2017 | clear renal cell carcinoma tissues  matched non-tumor tissues |
