# Supplementary material for: Circular RNA circPOLR2A promotes clear cell renal cell carcinoma progression by facilitating the UBE3C-induced ubiquitination of PEBP1 and, thereby, activating the ERK signaling pathway
Source: Mol Cancer. 2022 Jul 15;21:146. doi: 10.1186/s12943-022-01607-8 (PMC9284792; doi:10.1186/s12943-022-01607-8)
Supplement: Supplementary file 9 — Additional file 9: Supplemental Table 5. [file 12943_2022_1607_MOESM9_ESM.docx]

**Supplementary table 5: The information of primers, probes, shRNA and siRNAs**

| **Primers (5’ to 3’)** | | |
| --- | --- | --- |
| GAPDH | Forward | GGAGCGAGATCCCTCCAAAAT |
|  | Reverse | GGCTGTTGTCATACTTCTCATGG |
| U6 | Forward | CAGCACATATACTAAAATTGGAACG |
|  | Reverse | ACGAATTTGCGTGTCATCC |
| hsa_circRNA_092437 | Forward | GTCTTCCTGGAGCGGGTG |
|  | Reverse | GTCTGCATTGTACGGAGTTGTC |
| hsa_circRNA_105055 | Forward | TCCAGTGTGCTGATCTTCTGAC |
|  | Reverse | TGGAAGACCCGGAGTTGTTG |
| hsa_circRNA_100372 | Forward | GAGTGCAGGGGTCTCTCTTT |
|  | Reverse | CAACGTGTCAAGTGTCGGAT |
| hsa_circRNA_101965 | Forward | TACCTAGAGGGTCGGCAGCA |
|  | Reverse | GCGCACTGTATAGCCAACCTG |
| hsa_circRNA_103349 | Forward | CCAGCACTACCATGCCAATATG |
|  | Reverse | TGCCAGGGAAACACTGAGGA |
| hsa_circRNA_402565 | Forward | CAATCCCTCACATTCTCCA |
|  | Reverse | GTTGCCACAGTAACCACATC |
| hsa_circRNA_006562 | Forward | GTCGGATCAAAGAAGGCAGGA |
|  | Reverse | CCAGGTAGGGTTGCCACAGTA |
| hsa_circRNA_406752 | Forward | CCAGCACTACCATGCCAATATG |
|  | Reverse | TGCCAGGGAAACACTGAGGA |
| hsa_circRNA_406951 | Forward | GGACCATGCTCTTCCAGGTTC |
|  | Reverse | GGGTTTGGCATCCTCTTTCA |
| hsa_circRNA_092437  convergent primers | Forward | ATGGTTCCTCGCATGATTGTC |
|  | Reverse | GCACTGCTGTGAGTGTGTCCT |
| POLR2A mRNA | Forward | GCGGAATGGAAGCACGTTAAT |
|  | Reverse | CCCAGCACAAAACACTCCTC |
| PEBP1 mRNA | Forward | CCTCCACCGCTATGTCTGGC |
|  | Reverse | GCTGCTCGTACAGTTTGGGC |
| UBE3C mRNA | Forward | CTACCTTGTCACAGTCCG |
|  | Reverse | TGTCATCATTACAGTTTTGC |
| YTHDF2 mRNA | Forward | AGCCCCACTTCCTACCAGATG |
|  | Reverse | TGAGAACTGTTATTTCCCCATGC |
| METTL3 mRNA | Forward | CAAGCTGCACTTCAGACGAA |
|  | Reverse | GCTTGGCGTGTGGTCTTT |
| circPOLR2A-WT | Forward | GTCTTCCTGGAGCGGGTG |
|  | Reverse | GTCTGCATTGTACGGAGTTGTC |
| circPOLR2A-MUT | Forward | GCCATTGTTATCTTCAACCGG |
|  | Reverse | GCTGCATTGTACGGAGTTGTC |
|  |  |  |
| **shRNA and siRNAs (5’ to 3’)** | | |
| sh-circPOLR2A | TCTTCCTGGAGCGGGTGGA | |
| siPEBP1 | UAACGGGAAGUACAACUGG | |
| siUBE3C | GCCAGACAUUACUACUUCCUA | |
| siYTHDF2 | GCUCUGGAUAUAGUAGCAA | |
| siMETTL3 | GCCAAGGAACAAUCCAUUGUU | |
|  |  | |
| **Probes (5’ to 3’)** | | |
| circPOLR2A FISH probe:  GTGCCGTTCCACCCGCTCCAGGAAGA | | |
| Pull down circPOLR2A sense (circPOLR2A probe):  UAAUACGACUCACUAUAGGGAACUUGCACCUGCCACAGUCUCUGGAGACGCGAGCAGAGAUCCAGGAGCUGGCCAUGGUUCCUCGCAUGAUUGUCACCCCCCAGAGCAAUCGGCCUGUCAUGGGUAUUGUGCAGGACACACUCACAGCAGUGCGCAAAUUCACCAAGAGAGACGUCUUCCUGGAGCGGGUGGAACGGCACAUGUGUGAUGGGGACAUUGUUAUCUUCAACCGGCAGCCAACUCUGCACAAAAUGUCCAUGAUGGGGCAUCGGGUCCGCAUUCUCCCAUGGUCUACCUUUCGCUUGAAUCUUAGUGUGACAACUCCGUACAAUGCAGACUUUGACGGGGAUGAGAU | | |
| Pull down circPOLR2A antisense (Ctrl probe):  UAAUACGACUCACUAUAGGAUCUCAUCCCCGUCAAAGUCUGCAUUGUACGGAGUUGUCACACUAAGAUUCAAGCGAAAGGUAGACCAUGGGAGAAUGCGGACCCGAUGCCCCAUCAUGGACAUUUUGUGCAGAGUUGGCUGCCGGUUGAAGAUAACAAUGUCCCCAUCACACAUGUGCCGUUCCACCCGCUCCAGGAAGACGUCUCUCUUGGUGAAUUUGCGCACUGCUGUGAGUGUGUCCUGCACAAUACCCAUGACAGGCCGAUUGCUCUGGGGGGUGACAAUCAUGCGAGGAACCAUGGCCAGCUCCUGGAUCUCUGCUCGCGUCUCCAGAGACUGUGGCAGGUGCAAGUUC | | |
