# Supplementary material for: Circular RNA circPOLR2A promotes clear cell renal cell carcinoma progression by facilitating the UBE3C-induced ubiquitination of PEBP1 and, thereby, activating the ERK signaling pathway
Source: Mol Cancer. 2022 Jul 15;21:146. doi: 10.1186/s12943-022-01607-8 (PMC9284792; doi:10.1186/s12943-022-01607-8)
Supplement: Supplementary file 10 — Additional file 10: Supplemental Table 6. [file 12943_2022_1607_MOESM10_ESM.docx]

**Supplementary table 6: The information of antibodies**

| Antibodies | Source | Identifier |
| --- | --- | --- |
| PEBP1 | Beyotime | AF1906 |
| SMARCA1 | Cell signaling technology | 12483 |
| Ubiquitin | Cell signaling technology | 3936 |
| GAPDH | Abcam | ab8245 |
| RING1 | Abcam | ab180170 |
| UBE3C | Abcam | ab226173, ab243830 |
| TRIP12 | Abcam | ab86220 |
| Phospho-Erk1/2 | Cell signaling technology | 8544 |
| Erk1/2 | Cell signaling technology | 4695 |
| AGO2 | Cell signaling technology | 2897 |
| Ki-67 | Beyotime | AF1738 |
| Cleaved caspase-3 | Beyotime | AF1150 |
| YTHDF2 | Abcam | ab246514 |
