# Supplementary material for: Circular RNA circPOLR2A promotes clear cell renal cell carcinoma progression by facilitating the UBE3C-induced ubiquitination of PEBP1 and, thereby, activating the ERK signaling pathway
Source: Mol Cancer. 2022 Jul 15;21:146. doi: 10.1186/s12943-022-01607-8 (PMC9284792; doi:10.1186/s12943-022-01607-8)
Supplement: Supplementary file 11 — Additional file 11. Supplemental Methods and Materials. [file 12943_2022_1607_MOESM11_ESM.docx]

**Materials and methods**

1. Bioinformatics analysis

We downloaded the microarray datasets from GEO database (https://www.ncbi.nlm.nih.gov/geo/) with the keywords ‘circRNA’ and ‘kidney cancer’. We retrieved two datasets: GSE100186 and GSE137836. The probe information of platform GPL21825 was obtained from GEO database for further reference. The cRCC transcriptome and proteome data and clinical information were downloaded from TCGA (https://portal.gdc.cancer.gov/) and CPTAC (https://cptac-data-portal.georgetown.edu/) databases. R software (version 3.6.0) was utilized to conduct specific analysis.

2. Clinical samples and cell cultures

Totally, 32 pairs of cRCC tissues and matched adjacent normal tissues were collected from Jiangsu Cancer Hospital. Our study was approved by the Ethical committee of Jiangsu Cancer Hospital for using tissue specimens for research purposes. Prior informed consent was obtained from all of the patients. All cRCC specimens were staged according to the 2019 AJCC cRCC staging system. The cRCC patients with higher and lower circPOLR2A expression level than the median were allocated to the High and Low expression groups in Table 1, respectively.

Human RCC cell lines ACHN, Caki1, 786O and 769P and human renal proximal tubular epithelial cell line HK2 were purchased from the Chinese Academy of Science. Human RCC cell line Caki2 was purchased from the American Type Culture Collection (ATCC). All cell lines were cultured at 37°C in a humidified chamber with 5% CO2. All culture media contained 10% fetal bovine serum (FBS, Gibco). Specifically, we cultured 786O and ACHN cells in DMEN (Gibco), 769P cells in RPMI1640 (Gibco), and Caki1 and Caki2 cells in McCOY’s 5A (Gibco).

3. RNA and gDNA extraction

We extracted the total RNA from tissues or cells with Trizol reagent (Invitrogen) according to the manufacturer’s instructions. gDNA was extracted with a genomic DNA isolation kit (Sangon Biotech). Isolation of nuclear and cytoplasmic fractions was conducted with NE-PER Nuclear and Cytoplasmic Extraction Reagents (Thermo Fisher Scientific).

4. RNase R treatment, RNA reverse transcription and PCR

Total RNA was determined with a NanoDrop spectrophotometer (Thermo). If necessary, RNA was treated with 5U/μg RNase R (Epicentre Technologies) for 5min at 37 °C. RNA reverse transcription was conducted with SweScript RT I First Strand cDNA Synthesis Kit (Servicebio). We carried out PCR with GoTaq Green Master Mix (Promega) based on manufacturer’s instructions. Next, the PCR products were detected via electrophoresis and visualized with Safe Green (Biosharp). We performed the qRT-PCR with SYBR Green qPCR Master Mix (Servicebio). We normalized the expression of circRNAs and mRNAs with GAPDH. The sequences of primers were displayed in Supplemental Table 5.

5. Actinomycin D treatment, MG132 treatment and DZNep treatment

786O and Caki1 cells were incubated with 2μg/ml Actinomycin D (Sigma). Next, we collected cells at different time points. qRT-PCR was utilized to analyze the stability of circPOLR2A.

MG132 treatment was carried out by incubating cells with 20μM MG132 (MedChemExpress) for 12h. Subsequently, Co-IP and Western blot were performed to determine the protein level and ubiquitination.

DZNep treatment was conducted by incubating cells with 10μM DZNep (MedChemExpress) for 1h. Then, qRT-PCR was utilized to analyze the RNA expression.

6. FISH and immunofluorescence assay

In order to visualize the location of circPOLR2A and proteins, 786O cells were fixed. After permeabilizing and prehybridizing the cells, we performed the hybridization with Cy-3-conjugated circPOLR2A probes in the dark at 37°C overnight. Then, we rinsed the cells at 42°C in SSC buffer. The PBST containing 5% BSA was used as the blocking buffer. Subsequently, we incubated the cells with primary antibody at room temperature for 1h. The cells were incubated with secondary antibodies and DAPI. The confocal microscope was utilized to acquire the images. Antibody information was recorded in Supplemental Table 6.

7. Cell transfection

The lentivirus for circPOLR2A knockdown (sh-circPOLR2A) and circPOLR2A overexpression (p-circPOLR2A) was obtained from Genomeditech (Shanghai, China). The circPOLR2A-WT and circPOLR2A-MUT plasmids were synthesized by Genomeditech (Shanghai, China). The other siRNAs and plasmids were synthesized by GenePharma (Shanghai, China). The target sequences of shRNA and siRNAs were shown in Supplemental Table 5.

For stable transduction, 786O cells and Caki1 cells were transfected with lentivirus for circPOLR2A knockdown and overexpression on the basis of the manufacturer’s protocol. Next, we used the Puromycin or Hygromycin B to select stable cells. On the other hand, siRNAs and plasmids for PEBP1, UBE3C and ubiquitin were transfected via Lipofectamine 3000 kit (Invitrogen).

8. Cell proliferation assay, cell migration and invasion assay, and cell apoptosis assay

For Cell Counting Kit-8 (CCK-8, Dojindo Laboratories, Japan) assay, the cells were seeded into 96-well plates in triplicate. The cell viability was measured according to the manufacturer’s protocol. For colony formation assay, the cells were seeded into 6-well plates (500cells/well) in triplicate, and cultured for 10days. The colonies were fixed, stained and counted. For EdU assay kit (RiboBio, China), the cells were seeded into 24-well plates (3 × 10^4^cells/well) in triplicate. After cultured for 24h, the cells were incubated with 50μM EdU for 2h. Subsequent staining and visualization were carried out in accordance with the manufacturer’s instructions. The ratio of EdU-positive cells among total cells was calculated and analyzed in three random fields. Transwell inserts (Corning) with or without Matrigel (BD Biosciences) were utilized to perform migration and invasion assays according to the manufacturer’s protocol. The migrated/invaded cells were photographed and counted with the light microscope. Wound healing assay was utilized to evaluate the ability of cell migration. We seeded 3 × 10^5^ cells into 6‐well plates in triplicate. After cells grew to about 90% confluent, the scratch at the center of wells was made with sterile 200μl pipe tips. Cells were washed, cultured and photographed with the light microscope. For apoptosis assay, the Annexin V-FITC/PI Apoptosis Detection Kit (KeyGEN, China) were used to stain the cells based on the manufacturer’s manual. The apoptotic rate was determined via flow cytometry.

9. RNA pull-down and RIP assay

Following the manufacturer’s instructions of RNA pull-down kit (Bersinbio, China), the cells were harvested and lysed in lysis buffer. Then, the streptavidin-coated magnetic beads (Invitrogen) were incubated with the biotin-labeled circPOLR2A or Ctrl probes at 30°C overnight. We incubated the cell lysates with RNA probe-coupled beads, and collected the proteins pulled down using the elution buffer. Eventually, the retrieved proteins were further identified or validated by silver staining, MS analysis or western blot. The used probe sequences were displayed in Supplemental Table 5. The Magna RIP RNA-Binding Protein Immunoprecipitation Kit (Millipore, USA) was used to carry out the RIP experiment on the basis of the manual. And qRT-PCR was used to determine the co-precipitated RNA. The used antibody was listed in Supplemental Table 6.

10. Western blot and Co-IP

The total protein lysate was extracted with RIPA (Beyotime, China), and determined with BCA Protein Assay Kit (KeyGEN, China). The protein with equal amount was separated by 6-12% SDS-PAGE, and transferred on PVDF membranes. Following blocking, we incubated the membranes with primary antibody at 4°C overnight, then with secondary antibody at room temperature for 2h. Eventually, bands were visualized via the Bio-Rad ChemiDoc XRS system. For Co-IP assay, beads were preincubated with the antibody. Next, we lysed the cells with Co-IP buffer. After centrifugation, the supernatant was incubated with the beads at 4°C overnight. Finally, we washed the beads, added SDS buffer, and determined the immunoprecipitates by western blot. The information of used antibody was listed in Supplemental Table 6.

11. Tube formation assay

The cRCC cells with 70% confluency were washed with PBS, and cultured in the fresh media with 0.5% FBS for 24h. The media were then harvested as conditional media (CM). The CM were centrifuged, filtered through the 0.22μm filter, and finally stored at 4°C for use. HUVECs used in tube formation assay were pre-cultured for 12h in the CM. 50μl Matrigel (ABW, China) was added in each well of precooled 96-well plate, and solidified for 45min at 37°C. Next, we resuspended the pre-cultured HUVECs in CM. HUVECs were seeded in 96-well plates (1.5 × 10^4^cells/well), and cultured for 10h. The Calcein-AM dye (MCE, USA) was used to stain the HUVECs with the concentration of 6.25μg/ml for 30min at 37°C. Finally, the capillary-like structure was observed and photographed under the fluorescence microscopy (Axio Observer, Zeiss, German) (40 ×). Three randomly selected fields are collected for analysis. Total number of junctions and total vessels length analyzed by the AngioTool software (National Cancer Institute, USA) were used to assess the angiogenesis.

12. Animal models and IHC

For subcutaneous tumor growth model, 4-week-old male BALB/c nude mice were utilized. 5 × 10^6^ transfected cells were subcutaneously injected into the right side of each mouse. Tumor size was measured weekly. The following formula was applied to calculate tumor volume: Volume = Length × Width^2^ × 0.5. We euthanized all mice after 5 weeks. Then, tumors were collected, weighed and subjected to IHC staining. For the tail vein metastases model, 1 × 10^6^ transfected cells were injected into the tail vein of nude mice. The metastases were visualized via IVIS Spectrum in vivo imaging system. Finally, all mice were sacrificed after 8 weeks. And lung tissues were collected and further analyzed via H&E staining. For IHC, the retrieved tissues were fixed, embedded and sectioned. We incubated the slides with primary antibody at 4°C overnight, which was followed by incubation with secondary antibody for 1h at room temperature. We used the 3,3-diaminobenzidine (DAB) and hematoxylin to stain the slides. Eventually, three random fields on each slide were acquired for analysis.

13. MeRIP

The Magna MeRIP m6A Kit (Millipore) was utilized to examine the m6A modifications on circPOLR2A on the basis of the manufacturer’s instructions. Firstly, total RNA was sheared into approximately 100nt in length via metal-ion-induced fragmentation, which was followed by purification and incubation with beads conjugated with anti-m6A antibody or IgG at 4 °C overnight. Then, the beads were eluted via competition with free m6A. The RNeasy Kit (Qiagen) was used to recover the methylated RNA. We took 10% fragmented RNA as the input group, and conducted the qRT-PCR for further analysis.

14. Statistical analysis

We carried out all experiments at least three times and presented one representative experiment. The data are analyzed as mean ± standard deviation (SD). The statistical differences were assessed by Student's t-test or two-way ANOVA test. The clinicopathological features were analyzed by Fisher's precision probability test. The prognostic value was evaluated by Kaplan−Meier method, Log-rank test and cox regression analysis. All statistical analyses were performed with SPSS 20.0 software, R software (version 3.6.0) or GraphPad Prism (version 8.0). *p < 0.05; **p < 0.01; ***p < 0.001.
